# Supplementary material for: Electrophysiological development and functional plasticity in dissociated human cerebral organoids across multiple cell lines
Source: Cell Rep Methods. 2026 Mar 27;6(4):101371. doi: 10.1016/j.crmeth.2026.101371 (PMC13107059; doi:10.1016/j.crmeth.2026.101371)
Supplement: Document S2. Article plus supplemental information [file mmc4.pdf]

Cell Reports Methods

Article

Electrophysiological development and functional plasticity in dissociated human cerebral organoids across multiple cell lines

Graphical abstract

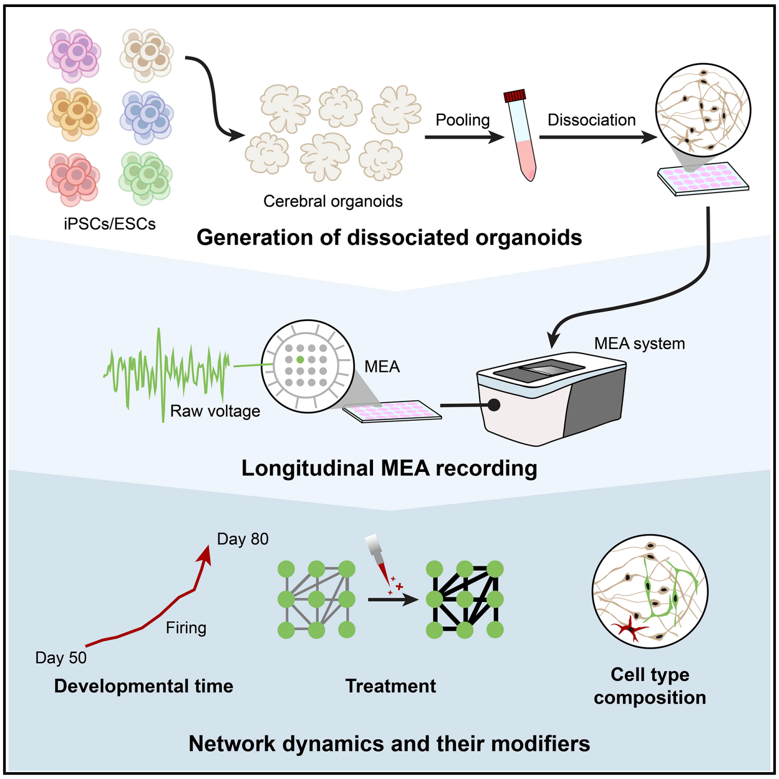

Authors

Adam Pavlinek, Sara Guerrisi, Kara O'Driscoll, ..., Madeline A. Lancaster, Anthony C. Vernon, Deepak P. Srivastava

Correspondence

ap2025@cantab.ac.uk (A.P.),
deepak.srivastava@kcl.ac.uk (D.P.S.)

In brief

Microelectrode array (MEA) recordings of human neural organoids allow for functional studies of brain network development, but these studies are currently limited in throughput. Pavlinek et al. use organoid dissociation to longitudinally study the development of neural networks and the effects of drug treatment, cell line, and cell-type composition.

Highlights

- Organoid dissociation enables scalable longitudinal electrophysiological studies
- Dissociated cerebral organoids develop functional activity and connectivity over time
- Variability in activity is linked to cell line and batch effects
- Multiple cell lines, batches, and analysis parameters should be used for MEA studies

Pavlinek et al., 2026, Cell Reports Methods *6*, 101371
April 20, 2026 © 2026 The Authors. Published by Elsevier Inc.
https://doi.org/10.1016/j.crmeth.2026.101371

Cell Reports Methods

CellPress
OPEN ACCESS

Article

Electrophysiological development and functional plasticity in dissociated human cerebral organoids across multiple cell lines

Adam Pavlinek,[1,2,*] Sara Guerrisi,[1,2,6] Kara O'Driscoll,[1,2,6] Lucia Dutan Polit,[1,2] Roland Nagy,[1,2] APEX consortium, Madeline A. Lancaster,[3] Anthony C. Vernon,[1,2,5] and Deepak P. Srivastava[1,2,4,5,7,*]

[1]Department of Basic and Clinical Neuroscience, Institute of Psychiatry, Psychology and Neuroscience, King's College London, London SE5 9RT, UK

[2]MRC Centre for Neurodevelopmental Disorders, King's College London, London, UK

[3]MRC Laboratory of Molecular Biology, Cambridge, UK

[4]Dementia Research Institute, King's College London, London, UK

[5]Senior author

[6]These authors contributed equally

[7]Lead contact

*Correspondence: ap2025@cantab.ac.uk (A.P.), deepak.srivastava@kcl.ac.uk (D.P.S.)

https://doi.org/10.1016/j.crmeth.2026.101371

MOTIVATION The electrophysiological properties of neural organoids remain incompletely characterized, particularly in scalable, high-throughput formats. Planar MEAs allow longitudinal recording from whole organoids but are limited to capturing surface activity and require adherence or intermittent recording, while more advanced 3D MEA systems are currently not yet widely available or scalable. We address this gap using a method of organoid-derived neuronal cultures for scalable MEA-based studies to systematically assess their network activity, connectivity, and responsiveness to physiological stimuli across multiple cell lines and developmental time points.

SUMMARY

Microelectrode arrays (MEAs) are increasingly used to profile the development of synchronized activity in neural organoids, yet no organoid study has reported on the consistency of electrophysiological development across cell lines. Here, we used dissociated neural organoids derived from six cell lines on MEAs to characterize functional synapse development using multiple parameters across time. The dissociated organoids demonstrated increasing functional connectivity and network activity over time across all cell lines and plasticity in response to synaptic-like stimulation. Like the organoids they were derived from, dissociated organoid cultures contained a diverse mixture of cell types. These results demonstrate that dissociated cerebral organoids can generate functional neurons, akin to primary neuronal cultures from brain tissue, providing a scalable model for studies of neurodevelopment and synaptic function. Consistent with unguided differentiation, we observed variability in activity parameters linked to donor cell line and batch effects, which must be considered in experimental design.

INTRODUCTION

Much of what is known about electrical activity in the developing human brain is inferred from rodent models, limiting our understanding of human-specific mechanisms that shape neural circuit function.[1] Despite evolutionary conservation,[2–4] the human cortex exhibits unique features distinguishing it from other primates and mammals.[5–10] Often originating in neurodevelopment,[10–12] these differences contribute to altered information processing and electrophysiological properties, including prolonged synaptic maturation and distinct patterns of neuronal excitability.[3,9,10,13–16] Recent advances in human-derived cellular models such as human induced pluripotent stem cell (iPSC)-derived neurons and neural organoids offer an opportunity to study the development of human neural networks and their emergent network properties.[17–19]

Micro-electrode arrays (MEAs) have widely been used to investigate the development and plasticity of two-dimensional (2D) human neural networks.[18–21] In monocultures of iPSC neurons, initially random spiking and bursting can gradually become

© 2026 The Authors. Published by Elsevier Inc.
This is an open access article under the CC BY license (http://creativecommons.org/licenses/by/4.0/).

Table 1. Advantages and limitations of whole and dissociated organoid MEA recording approaches

	Advantages	Limitations	Applications
Whole organoids	• recording straightforward to set up<br>• spontaneous assembly of neuronal networks in 3D	• regular longitudinal recording:<br>A) with attachment encourages 2D neuronal outgrowth, necrotic core, maintaining electrode contact is challenging<br>B) without attachment requires manipulation; different neurons and regions of the organoid are recorded<br>• surface recording or limited penetration of electrodes into organoid with current widely available MEA plates and grids	• low-throughput experiments<br>• single-time-point recordings<br>• assessment of organoid health and activity<br>• assessment of spatial activity patterns, such as in assembloids
Dissociated organoids	• 2D layer allows for direct contact with electrodes<br>• pooling of multiple organoids to reduce variability or straightforward combination of cells from different protocols<br>• reduced variability in morphology and structure<br>• longitudinal recordings	• loss of 3D spatial organization<br>• dissociation disrupts existing connections between neurons<br>• cells are less dense than in organoid<br>• cells that detach cannot be re-attached	• high-throughput drug and genetic screening<br>• longitudinal experiments<br>• combination with other 2D assays, such as live imaging, calcium imaging, patch-clamping, custom chip setups
Shared across both	• undergo initial patterning and development in a 3D context<br>• contain diverse cell types	• more variable than monocultures	• modeling neurodevelopment

more complex,[18] with emergence of properties such as network bursting, and oscillations, indicative of synaptic connectivity.[20,22–24] Such spontaneous neuronal activity is thought to aid the development of synchronized neuronal activity, refinement of synaptic connections, and network maturation.[25,26] As well as investigating normative development, MEA recordings can be used to study disease-specific phenotypes,[18] such as using hiPSC-derived neurons generated from autistic individuals.[27–29] However, while 2D monocultures are amenable to electrophysiological analysis, they lack the cellular diversity of the developing human brain, with some methods requiring rodent astrocytes.[18,19]

Three-dimensional (3D) human organoid models offer a more physiologically relevant platform for studying human-specific neurodevelopment.[30] Specifically, transcriptomic analyses across different differentiation methods have shown that major gene expression programs, cell types, and epigenetic regulation in the developing brain are better preserved in 3D organoids when compared to human fetal tissue, than in 2D cultures,[31–36] at least up until the late second trimester of fetal development.[37] This includes human-specific developmental changes, such as delayed neuronal maturation and human-specific gene expression.[8] Current protocols for generation of neural organoids can be categorized as either unguided (relying on intrinsic tissue patterning) or regionalized (guided by exogenously added factors).[33,38] While unguided neural organoids have increased variability between organoids as compared to more guided approaches, they also have greater cellular diversity.[39]

Human neural organoids display spontaneous electrical activity and develop synchronized network activity, measurable using calcium imaging, patch-clamp recordings,[40,41] and MEAs.[42–45] These studies have typically characterized the neuronal networks in organoids and their dynamics over time in limited numbers of organoids from $N = 1$ or 2 donor cell lines,[41–43,46] despite organoid differentiation being known to be cell line dependent.[47] No study has systematically studied the effects of organoid batch, cell line, and chromosomal sex on the development of electrophysiological activity, as measured by MEAs, in unguided organoid models. Understanding batch and donor line variability in MEA readouts is essential for informing the design and interpretation of studies that use patient-derived or genetically engineered lines. Additionally, while there is evidence that organoid electrical activity changes in response to drugs, such as tetrodotoxin[44] or diazepam,[48] the effects of more physiological stimuli, such as those for induction of long-term potentiation[49] or steroid hormones have not been investigated.

In this study, we present organoid dissociation and 2D culture as a scalable approach for recording of activity over longer time periods (Table 1). Unlike neuronal monocultures, dissociated organoid cultures benefit from the increased cell-type diversity of organoids and undergo initial patterning and development in a 3D context. We systematically characterize the development of spontaneous electrophysiological activity and network properties in dissociated unguided cerebral organoids derived from six donor cell lines. By leveraging MEA recordings, we assess how batch effects, donor cell line, and chromosomal sex influence network maturation over time. We further evaluate the responsiveness of dissociated organoid-derived networks to chemical

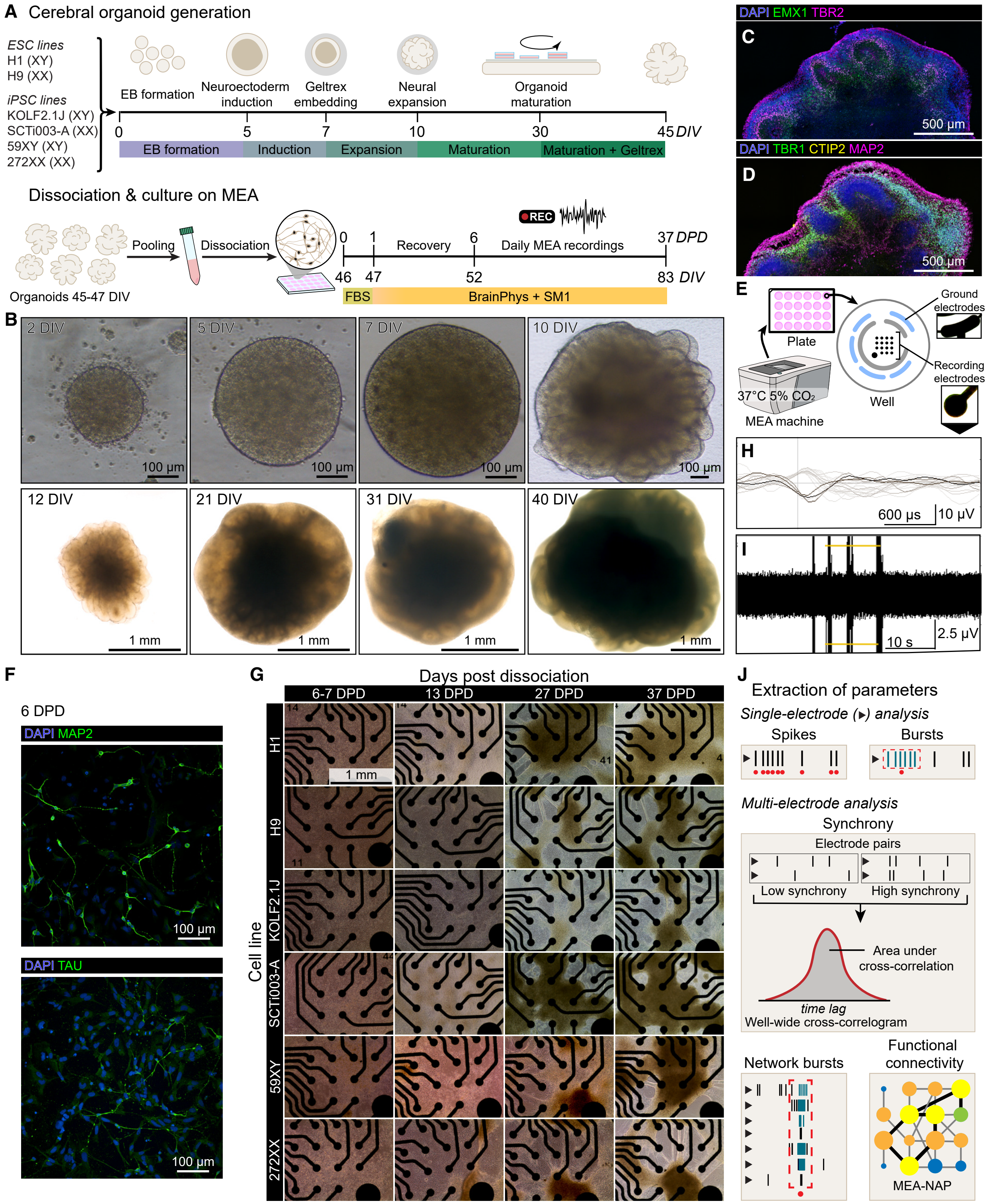

Figure 1. Overview of dissociated organoid culture, morphology, and MEA recording

(A) Organoid generation and dissociated culture timeline. Top: generation of cerebral organoids with days *in vitro* (DIV). Bottom: organoid dissociation and culture. Timing from the day of dissociation shown as days post-dissociation (DPD). Colored bar below timelines indicates culture medium used.

(legend continued on next page)

long-term potentiation (chLTP) induction[49] and the steroid hormone 17β-estradiol (E2), a key modulator of synaptic plasticity in rodent models.[50–56] We show that despite batch-dependent variability, dissociated organoid cultures exhibit consistent temporal progression in network properties, supporting their use as a scalable and reproducible model for studying human neural network activity.

RESULTS

Dissociation of cerebral organoids and culture on MEAs

To analyze the development of electrical activity over time in neural cultures from dissociated organoids, we generated unguided neural organoids from two ESC and four iPSC cell lines and cultured them up to 45 days *in vitro* (DIV) using the established "cerebral organoid" method[44,57,58] (Figure 1A). We used organoids with visible cortical plate regions (Figure 1B) from batches that contained organoids with dorsal forebrain tissue (Figure 1C), using established characterization markers.[58–60] At 40 DIV, cerebral organoids have early-born MAP2-positive neurons on the outside of neuroepithelial regions, which in dorsal forebrain regions were positive for TBR1 and CTIP2 (Figure 1D). Random spiking was detected in a subset of whole 40 DIV organoids attached to an MEA (Figure S1) but with no detectable network activity. We pooled multiple organoids together, dissociated them into single cells, and plated them onto multi-well MEA plates (Figure 1E). At 6 days post-dissociation (DPD), the cells had visible processes and were positive for neuronal markers (Figure 1F). Across dissociated cultures from the six cell lines, we observed that cells were initially evenly distributed in the well but became more aggregated over time (Figure 1G): At 13 DPD, the cells had visible processes but also some early signs of uneven aggregation, with denser areas of cells in the well. By 27 DPD, there was extensive clustering of cells, with bundles of axons extending between clusters; the clusters then increased in size between 27 and 37 DPD. The dissociated organoid cultures developed spontaneous activity and clustered bursting within 20 DPD (Figures 1H and 1I).

Dissociated organoids from six donor cell lines develop network connectivity over time

To determine the dynamics of electrophysiological activity over time in the dissociated organoid cultures, we analyzed changes in activity from six DPD, when we could detect spiking activity, to 37 DPD, focusing on six core parameters (Figure 1J; Table S1). The core parameters, selected *a priori*, together describe firing, synchronized activity, and network connectivity in each well. These core parameters were *mean firing rate (MFR)* as a measure of firing, *number of bursts* as a measure of single-electrode bursting, *area under normalized cross-correlation (AUNCC)* as a measure of synchrony of spiking across different electrodes,[61] *network burst number (NBN)* as a measure of whole-well bursting, *mean node degree (MND)* as a measure of connectivity, and *edge weight* as a measure of the strength of connectivity (Table S1). We generally observed increases in firing rate and bursting over increasing DPD (Figure 2A, Video S1); however, this varied between batches and cell lines. For instance, MFR was increased at 37 DPD compared to 6 DPD in H9, KOLF2.1J, and 59XY line dissociated organoids, but we did not observe a significant increase in the H1, SCTi003-A, and 272XX lines (Figure 2A). We saw more consistent increases in connectivity over time, including increasing AUNCC, as well as increasing MND. Both MND and AUNCC were higher at 37 DPD compared to 6 DPD across all lines (Figures 2B and 2C; Table S3). Correspondingly, regular network bursts occurred in dissociated organoids from all cell lines (Figure 2D, Video S1). All spiking was abolished upon tetrodotoxin exposure (Figures S2G and S2H). Overall, we saw increasing number and strength of connections over time and greater participation of nodes in the network (Figure 2E). However, there was variability between wells in these parameters (Figures 2F–2H). For example, only a subset of wells developed network bursting in H1 and SCTi003-A cultures (Figure 2H). There were cell line and batch differences; cells from the KOLF2.1J line developed the highest AUNCC and NBN, followed by H9 (Figures 2G and 2H). For the six lines we assessed, there was a difference between XX and XY lines in two of the six core parameters at 37 DPD (Table S3), with XY lines having on average a greater NBN and edge weight compared to XX. ESC networks had lower node degree (Table S3) compared to iPSC networks, but we did not see iPSC/ESC line differences in the other core parameters. Analysis of variance testing showed statistically significant variation across all core parameters with respect to cell line at 37 DPD (Table S3). Taken together, these results show that dissociated

(B) Brightfield images of organoid development hallmarks: embryoid body (EB) stage (0–5 DIV), neural induction (7 DIV), expansion of neuroepithelial buds following Geltrex embedding (10 DIV), and organoid maturation (12 DIV–40 DIV). Different representative H9 line organoids are shown.

(C and D) Composite images of neural rosettes from tile scans of two adjacent serial sections of a representative SCTi003-A line organoid at 40 DIV. Immunostaining for EMX1 and TBR2 (D), MAP2, CTIP2, and TBR1 (E). Brightness and contrast adjusted for clarity. See also Figure S1.

(E) Schematic of recording setup with 24-well MEA plate showing within-well arrangement of electrodes.

(F) Representative fluorescent images of neuronal markers MAP2 and TAU at 6 DPD, dissociated H9 line organoids.

(G) Representative brightfield images of dissociated organoids on 16-electrode MEA. Each column shows 6-7, 13, 27, and 37 DPD. Each row shows images from one batch of each cell line. All images have the same scale.

(H) Snapshot of recording from H9 dissociated organoids at 34 DPD showing unsorted overlaid spike waveforms recorded at a single electrode. Waveforms correspond to spikes above the 5.5 standard deviation (SD) threshold. The darkest waveform represents the most recent spike recorded at the electrode. Scale bar represents voltage (vertical) and time (horizontal).

(I) Representative time segment at 34 DPD showing raw neural spikes recorded at one electrode, in the same well and time as in (G). Horizontal lines indicate the 5.5 SD threshold. Scale bar represents voltage (vertical) and time (horizontal).

(J) Schematic of key single electrode and multi-electrode neuronal activity parameters. Parameter details in Table S1. MEA-NAP, MEA network analysis pipeline.[24]

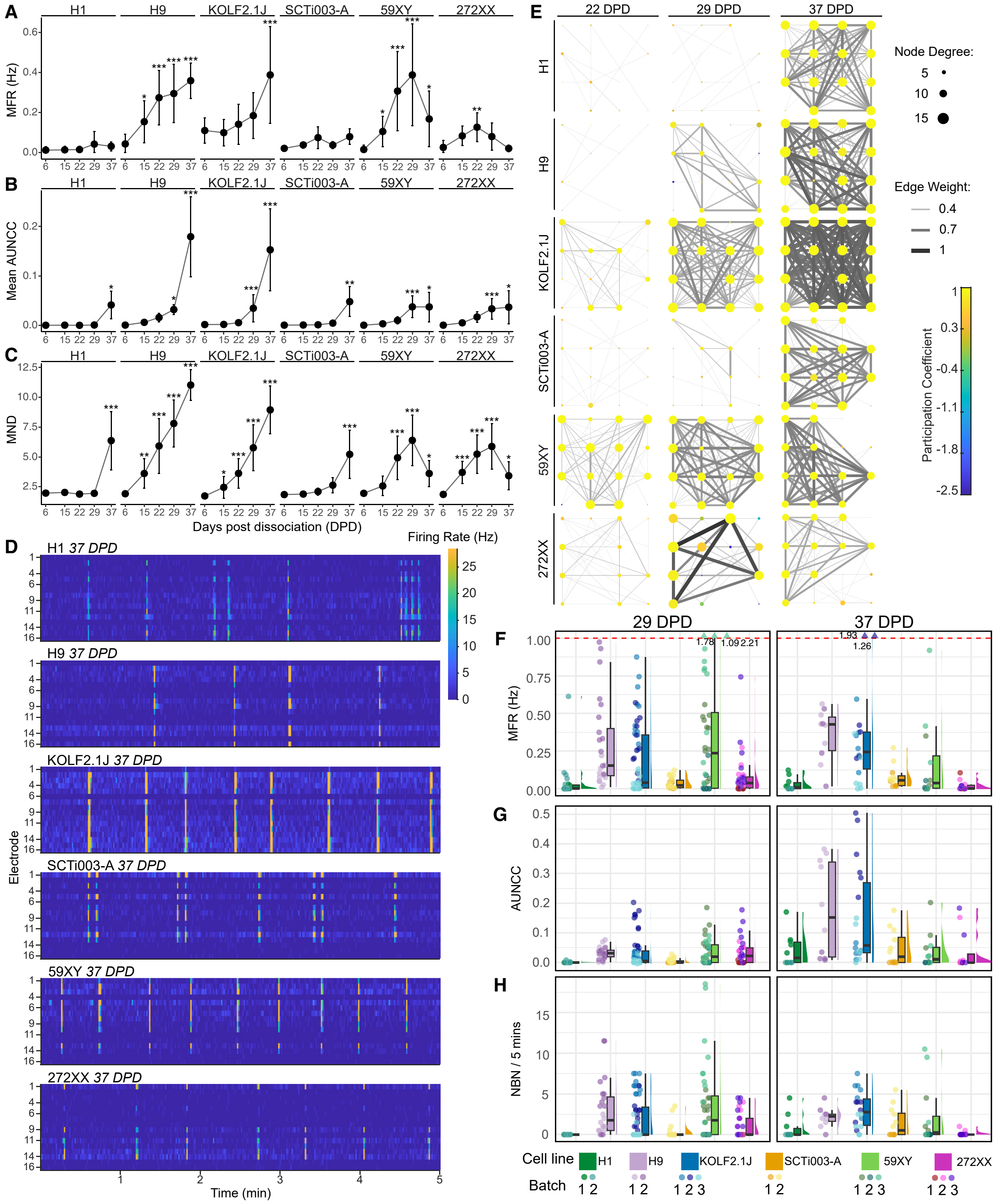

Figure 2. Dissociated organoids from six cell lines develop network connectivity over time

(A–C) Line graphs showing well averages of firing rate (MFR), area under normalized cross-correlation (AUNCC), and node degree (MND) at 6, 15, 22, 29, and 37 DPD. Points represent the mean of well averages ±SD. *$p < 0.05$, **$p < 0.01$, ***$p < 0.001$ (6 vs. 37 DPD). Mixed-effects model with estimated marginal means post-hoc comparisons (Bonferroni correction). Full statistics in Table S3. $N \geq 12$ wells per line, time point, and batch across all lines, across two (H1, H9,

(legend continued on next page)

organoid networks possess inherent variability arising from different organoid batches and donor lines, but all show a significant increase in network activity over time.

Dimensionality reduction of neuronal and network parameters shows temporal and batch effects in dissociated organoid electrical activity

To integrate electrophysiological and network features across parameters, we visualized data from individual culture wells, across all cell lines, for 13 parameters, using a uniform manifold approximation and projection (UMAP) approach across five time points. The UMAP visualization revealed clustering of wells based on time point and batch (Figures 3A and 3B). Wells with similar activity profiles clustered together, with a progression from low to high firing, burst percentage, bursting, and mean number of significant connections (Figures 3C–3F). Separately, we used unsupervised Euclidian clustering to identify clusters based on similarity between parameters (Figures 3G–3I). This showed two main clusters among the six highest level clusters, where one smaller cluster corresponded to active wells with more mature networks at later time points, while the largest cluster consisted of less active wells or wells at earlier time points (Figure 3J). The proportion of wells in the more active clusters increased with culture time (Figure 2G). Within these clusters, there were subclusters of wells from the same cell line, suggesting cell line effects. The proportions of wells from each line in each cluster differed by cell line, with KOLF2.1J having greater activity and proportion of wells in the more active cluster at 29 DPD. We further analyzed batch and cell line effects using principal-component analysis (PCA) (Figures 3K–3S). Across time points, there was a gradient of wells according to age along PC1 and PC2; however, some older, less active wells overlapped with earlier time points, and there was no clear clustering by line (Figures 3K and M). Outliers generally consisted of older, more active wells. We focused on the 37 DPD time point to assess batch effects. PCA at 37 DPD suggested consistency across batches and cell lines for most experiments (Figure 3N). We saw batch effects within lines, such as for SCTi003-A and H9, where wells from different batches clustered separately (Figure 3S). For instance, batch (1) of SCTi003-A was less active, and the wells primarily clustered with each other, while wells from batch (2) of SCTi003-A were more active and clustered with most of the wells from the other cell lines. These results suggest that while batch effects and random differences in networks between wells contribute to variation in dissociated organoid electrophysiological activity and maturation, analysis across multiple parameters also reveals consistency in activity across batches and cell lines.

Development of spontaneous electrical activity and network properties vary between batches

To further characterize batch and temporal effects in dissociated organoids, we analyzed the dynamics of acquisition of network activity across three batches of KOLF2.1J-dissociated organoids with daily recordings (Figure 4), as this line has been suggested as a reference line across studies.[62] Spontaneous activity was present across all three KOLF2.1J batches, but network bursting only developed over time in two of the batches (Figure 4D). Batch differences were present not just in the absolute level of firing and network bursting but also in the dynamics over time (Figures 4A–4D). While network bursting and synchrony, if at all present, increased with time after ~15–20 DPD, bursting and spike parameters had a more complex pattern. In one batch, there appeared to be two peaks of high firing, with a high MFR and burst activity early after dissociation (~10 DPD) and a second peak past ~25 DPD. In the batch where network bursting did not occur, there was also an early peak in firing at ~10 DPD. In this batch, spontaneous firing decreased over time but started out higher than in another batch that did develop network activity (Figures 4A and 4E). The differences in overall activity levels were not simply due to different numbers of active electrodes, as weighted MFR was still the lowest in batch 3 and highest in batch 1 (Figure S4).

Interestingly, we also observed differences in the characteristics of network burst events. The batch with the highest AUNCC after 25 DPD (batch 2, Figure 4C) had less frequent network bursting (Table S2). The differences in synchrony suggested differences in the characteristics in network burst events, which we further characterized at 30 DPD (Table S2). This difference in network burst characteristics was also apparent when comparing raster plots of activity from representative wells (Figures 4I and 4K). Other parameters were more consistent over time between the three batches (Figure S4), and the variation between wells in these parameters (Figure 4) was similar between the two batches that had synchronized network activity (Figure S4). Some parameters of network activity were similar between the two batches with network activity, such as network inter-burst interval (IBI) coefficient of variation, burst percentage, network burst duration, and median/mean ISI within network bursts (Figures 4M–4P).

SCTi003-A) or three (KOLF2.1J, 59XY, 272XX) batches. At 37 DPD, the N is halved as only control wells are shown; see STAR Methods for details. See also Figure S2.

(D) Raster plots at 37 DPD of representative wells with network activity for each cell line showing relative MFR in hertz (Hz, color bar) in 1-s bins for each electrode (rows) over 5 min scaled to the MFR range in the entire dataset. See also Video S1.

(E) Scaled network plots showing functional connectivity at 22, 29, and 37 DPD, corresponding to the wells shown in (D). The nodes (circles) represent neuronal activity at each electrode in the spatial arrangement of the MEA. Node color represents the participation coefficient. Node degree (size of circle) represents the number of functional connections with other nodes. Edges (lines) represent significant functional connections between nodes, and edge weight (line thickness) represents strength of connectivity. Participation coefficient color bar, node size, and edge thickness are scaled to the entire dataset.

(F–H) Box (median and interquartile range, IQR) and violin plots of the MFR (F), AUNCC (G), and network burst number (NBN)/5 min (H) per well at 29 and 37 DPD. Points represent well averages across 5 min of recording. Point colors indicate the batch and cell line. Boxplot color indicates cell line, from left to right: H1, H9, KOLF2.1J, SCTi003-A, 59XY, and 272XX. For clarity, MFR in (F) is capped at 1 Hz (dotted line); points >1 Hz are annotated with actual values. See also Figure S2.

CellPress
OPEN ACCESS

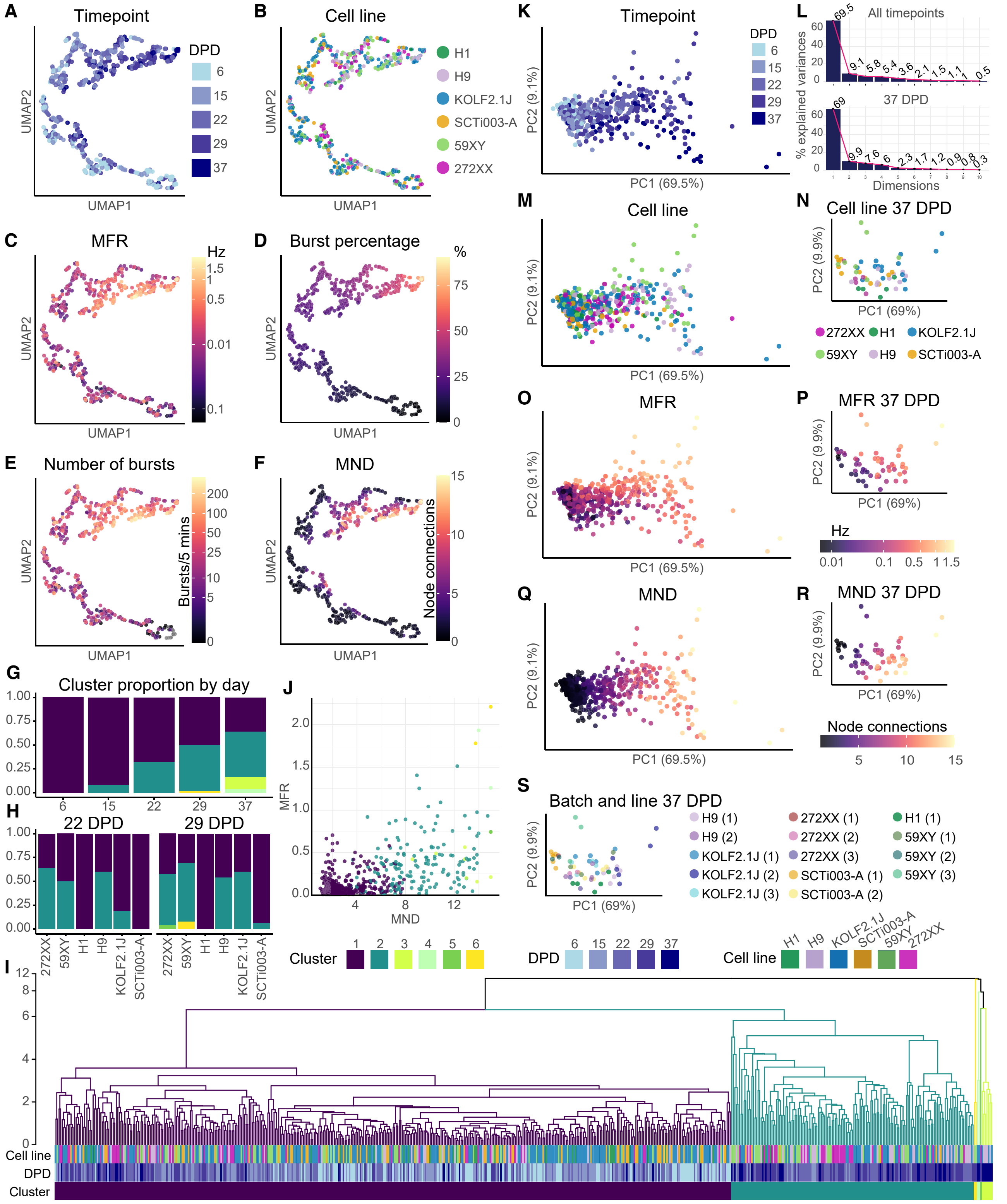

(legend on next page)

Overall, dissociated organoids show clear batch differences at the level of individual parameters, with some parameters, such as firing rate, showing greater variability than others.

Dissociated organoid culture morphology, cell type, and regional identity are potential contributors to variation in MEA parameters between batches

Given the variability in spontaneous activity between different cell lines and batches, we predicted that this difference may be due to differences in cell and regional identity composition between experiments. We used immunocytochemistry of cells plated from the initial dissociation grown in parallel to the MEA experiments and bulk RNA sequencing (RNA-seq) following the final recording at 37 DPD to characterize the degree of variation in regional identity and cell-type composition in the dissociated cultures (Figure 5). We qualitatively compared cells from dissociated organoids from a highly active batch (KOLF2.1J batch 2), medium active batch (SCTi003-A batch 2), and less active batch (H1 batch 1), (Figures 5A–5C). We stained cells for markers of excitatory and inhibitory neurons, glia, and dorsal forebrain.

The dissociated organoids consisted of a mixture of cell types, with star-shaped GFAP-positive cells, MAP2-positive cells with a neuronal morphology, of which some nuclei were positive for excitatory markers CTIP2 and NEUROD2, or inhibitory marker DLX2 (Figures 5A and 5B). These neurons were found in joint clusters. In addition, there were processes with distributed puncta of VGLUT1 and separate, non-overlapping GABA-positive processes (Figure 5C), which appeared to radiate out of the same neuronal clusters. There were some clear differences in composition in the dissociated culture from H1 organoids, with what morphologically appeared to be an increased number of progenitors, increased number of GFAP-positive cells, and more diffuse and less intense EMX1 expression (Figures 5A and 5B). The H1 culture contained fewer MAP2-positive neurons and appeared denser in terms of overall number of DAPI-positive nuclei.

Given the variability in activity between wells in the same experiment, we hypothesized that well-to-well differences in spontaneous activity may also be due to the random differences in the sizes and positioning of clumps in different wells that developed over time. We quantified the degree of clustering in different wells as a cluster ratio of sparse to dense areas and plotted this against firing rate at active electrodes (Figure 5D). There was a significant positive correlation, where the sparser the well, the greater the MFR was ($R = 0.57$, $p = 0.001$). However, this appeared to be cell line or batch dependent. Batches with high cell density had very low firing rates, but batches with clustering and sparse areas had more variable firing rates. We did not see significant correlation of cell clustering with bursting, network bursting, or AUNCC, suggesting that network characteristics are less dependent on cell density than firing rate. There were still clear contributions of cell line and batch (Figure 5D).

Deconvolution of bulk RNA-seq data[64] showed that most dissociated organoid samples were estimated to contain cells representing pallium, in varied amounts. Some samples showed reduced pallial representation with other brain regions, including pontine hindbrain, subpallium, midbrain, and hypothalamus, present at varying proportions (Figure 5G). Notably, 272XX- and H1-dissociated organoids, which had the lowest firing rates, were estimated to primarily consist of prepontine hindbrain cells. Both batch 1 of SCTi003-A and batches 2 and 3 of 59XY, which clustered separately at 37 DPD based on MEA activity parameters (Figure 3S), also showed increased proportion of hypothalamus (Figure 5G). To further characterize the cell-type composition of the dissociated organoid samples, we examined cell-type marker expression (Figure 5H). Consistent with the immunocytochemistry data, we found that the H1 line culture had altered levels of cell type markers, such as increased GFAP expression. Hierarchical clustering based on the selected cell-type markers mirrored PCA clustering based on the top 500 most differentially expressed genes between the samples (Figure 5H).

This analysis suggests that differences in electrical activity parameters parallel differences in cell identity and cell type composition, which can be detected at both the protein and gene expression levels.

Dissociated organoids respond to a chemical plasticity stimulus

To test whether the neurons in the dissociated cultures showed plasticity in response to a stimulus, we used a chemically induced LTP-like (chLTP) stimulation paradigm[49] (Figure 6A). Thirty minutes post-chLTP treatment, there was an initial decrease in spontaneous firing. Three hours post-treatment, MFR, number of bursts, AUNCC, and NBN increased relative to baseline in chLTP-treated wells (Figure 6). The greatest chLTP response occurred between 6 and 24 h before diminishing. Six hours post-treatment, there was a significant increase in MFR, AUNCC, number of bursts, and network bursts in the chLTP condition relative to vehicle (Table S3). There was an increase

Figure 3. Dimensionality reduction of neuronal and network parameters shows temporal and batch effects in dissociated organoid electrical activity

(A–F) Uniform manifold approximation and projection (UMAP) visualizations of MEA wells from 14 neuronal activity and network parameters showing all wells (points) with active electrodes at 6, 15, 22, 29, and 37 DPD. Color-coded by time point (A), cell line and batch (B), MFR (log10 color scale) (C), burst percentage (D), number of bursts (log10 color scale with 0 shown as black) (E), and MND (F). See also Figure S3.

(G–I) Hierarchical cluster analysis of wells with active electrodes across five time points based on 14 scaled MEA parameters. (G) Bar chart showing proportion of wells assigned to clusters by time point. (H) Bar charts of the proportion of wells assigned to each cluster at 22 DPD and 29 DPD, grouped by cell line. (I) Dendrogram of all wells; branch length indicates degree of similarity in activity. Dendrogram colored by top six identified clusters (k = 6). Color bars (bottom) represent well cluster, cell line, and DPD.

(J) Scatterplot of mean node degree and MFR for all wells with active electrodes across five time points colored by assigned cluster; each point represents the well average at one time point.

(K–S) Principal-component analysis (PCA) of 14 MEA parameters for wells with active electrodes at five time points and 37 DPD. PCA plot colored by cell line (M and N), MFR (log10 color scale) (O and P), mean node degree (Q and R), and batch (S). Each point in (K and M–S) represents one well. See also Figure S3.

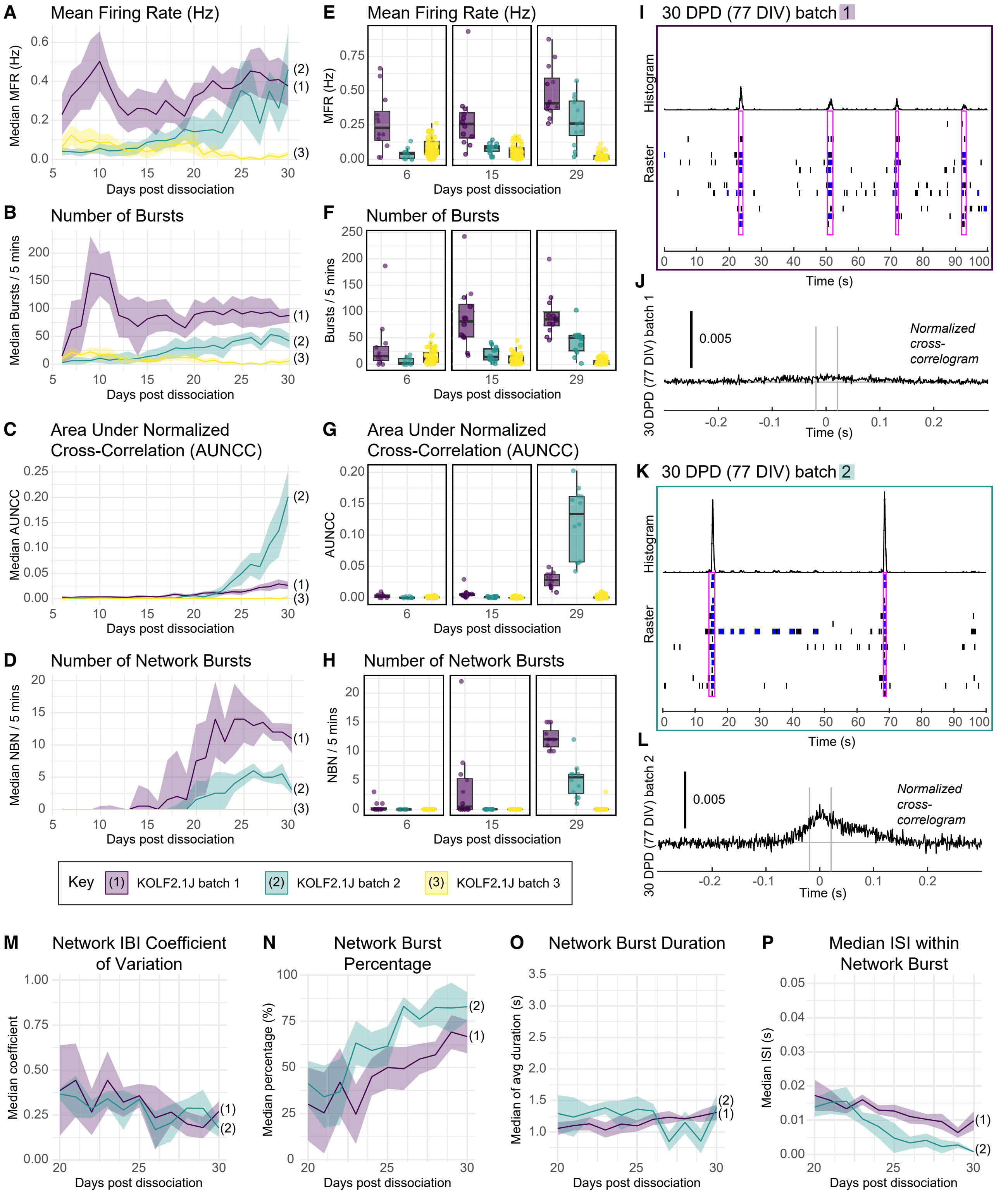

Figure 4. Development of spontaneous electrical activity and network properties vary between batches

(A–D) Line graphs showing the median firing rate (A), number of bursts (B), AUNCC (G), and NBN (H) across three batches of dissociated KOLF2.1J organoids, with one time point per day. The line represents the median of all well averages (*N* = 12 for batch 1 and 2 and *N* = 24 for batch 3). The ribbon represents the IQR. Colors indicate batch.

(legend continued on next page)

in single-electrode bursting and synchrony, with shorter bursts compared to baseline measurements or the vehicle condition (Figures 6B and 6C). While there was no significant increase in MND or edge weight, we observed increased strength of network connections in more active wells (Figure 6D). PCA across the six core parameters showed a clear shift in the overall activity profile of chLTP-exposed wells; at baseline, wells from both conditions clustered together (Figure 6E), while at 6 h post-chLTP, there was separation of wells based on whether they were treated with chLTP or vehicle (Figure 6F). The magnitude of response to chLTP differed between replicates and wells (Figures 6G–6L). Across both replicates, median single-electrode measures of activity, firing rate, and number of bursts increased, although the increase was larger in the first replicate. In contrast, synchrony and NBN increased minimally in the second replicate (Figures 6I and 6J). In wells with bursting, there was a consistent decrease after chLTP in the variability of the inter-spike interval (ISI), meaning increased consistency of firing, and a reduction in the IBI, meaning more regular bursting. These two parameters clearly separated the vehicle and chLTP conditions (Figures 6K and 6L). Thus, despite variability in responses between wells and replicates, dissociated organoids show plasticity in response to stimulation, with chLTP being a reproducible method for increasing network activity in dissociated organoids.

17β-estradiol does not affect electrical activity in dissociated organoids

Given the known role of 17β-estradiol in modulating synapses in rodents,[50–56] and iPSC-derived neurons,[65–68] we tested the effect of E2 in combination with chLTP. Overall, we did not detect a significant effect of E2 (Figure S5; Table S3). In line with this, E2 pre-treatment did not determine PCA clustering (Figure S5E). Hierarchical clustering on the PCA data showed that all but one of the samples without chLTP clustered together (Figure S5F). The chLTP-treated samples clustered into three groups, presumably based on the magnitude of response to chLTP.

To determine whether longer-term E2 exposure affects spontaneous electrical activity in dissociated organoids, we added 10nM E2 or vehicle daily for 7 days (Figure S6). There was no consistent effect of E2 exposure across the three cell lines tested. There continued to be significant cell line and batch differences and changes over time in parameters such as NBN, as reflected in PCA across all wells (Figure S6). Together, our analysis of dissociated organoids acutely exposed to chLTP, chLTP with E2, E2 alone, or exposed to chronic E2 showed that while chLTP induced changes in spontaneous activity across multiple parameters, E2 did not appear to have an effect under the conditions tested.

DISCUSSION

Neuronal networks have been characterized in limited numbers of organoids and donor lines.[41–43] Building on existing studies, we characterized the development of neural networks specifically using unguided dissociated organoids, showing scalability to multiple cell lines and batches. We observed a gradual development of activity over time in the dissociated organoids, in line with other studies performed with 2D and 3D neuronal cultures.[21,42,43,69] Previous studies have observed more complex activity generally arising with longer culture periods. Studies using organoid slices have shown long distance connectivity at 130 days of culture,[44] while whole unguided organoids had network bursting at 120 days, with weak activity at 1 month of culture.[43] In our study, dissociated organoids developed spontaneous network activity much earlier across several cell lines and batches, with network bursting in some replicates occurring after around 20 DPD. This is similar to previously described synchronized calcium bursting at 30 DPD in dissociated organoid cultures.[41]

In a recent study using homogeneous monocultures of iPSC neurons from multiple lines, Mossink et al.[18] saw subtle variability between wells and experiments and no line-specific differences, recommending a minimum of two replicates per line. With our more complex system, we observed a hierarchy of variability at the level of cell line, batch, and variability between neural networks in each well. Like Mossink et al.,[18] we observed variability in some parameters but not others, including similar network burst duration and similar magnitudes of within-batch variability for parameters such as IBI. We suggest that care needs to be taken in selection of MEA parameters for analysis. For example, in line with others,[18] we found that MFR was highly variable and dependent on neuronal density. Although neuronal clustering affected firing rate, the same phenomenon has been observed in other dissociated organoid culture studies[41] and may accelerate the development of network activity.[70] Our integration of multiple parameters using a dimensionality reduction approach, as others have done with neuronal monocultures,[18] may be a useful method for extracting electrophysiological phenotypes while avoiding the noise associated with looking at single parameters like MFR.

Single-cell RNA sequencing of organoids has demonstrated a high degree of consistency in cell-type composition between individual guided organoids and batches,[31,71] while unguided

(E–H) Boxplots of the min, max, median, and IQR of the MFR (E), number of bursts/5 min (F), AUNCC (G), and NBN/5 min (H) per well at three different time points (6, 15, and 29 DPD), KOLF21.J line. Points represent well averages across 5 min of recording. Colors indicate batch.

(I–L) Comparison of two KOLF2.1J dissociated organoid batches with network activity. Raster plots from one representative well of each batch (I, K) showing spikes recorded across 100 s at 30 DPD (77 DIV), single-electrode bursts (blue), and network bursts (pink boxes). Normalized well-wide cross-correlograms (J and L) for the corresponding wells in (I) and (K) are shown below. The cross-correlogram shows a pooled measure of the relatedness of spike trains across all possible electrode pairs in the well. A higher peak indicates that spikes from electrode pairs occur closer together in time. The time axis indicates the time lag from 0 for each spike in a paired electrode relative to the reference spike in the corresponding electrode, across all electrode pairs. The gray lines indicate the synchrony window.

(M–P) Line graphs of parameters for two KOLF2.1J dissociated organoid batches with network activity, showing the median of all well averages for network inter-burst interval (IBI) coefficient of variation (M), network burst percentage (N), network burst duration (O), and median/mean inter-spike interval (ISI) within network bursts (P) between 20 and 30 DPD. The ribbon shows the IQR. N = 12 wells per batch.

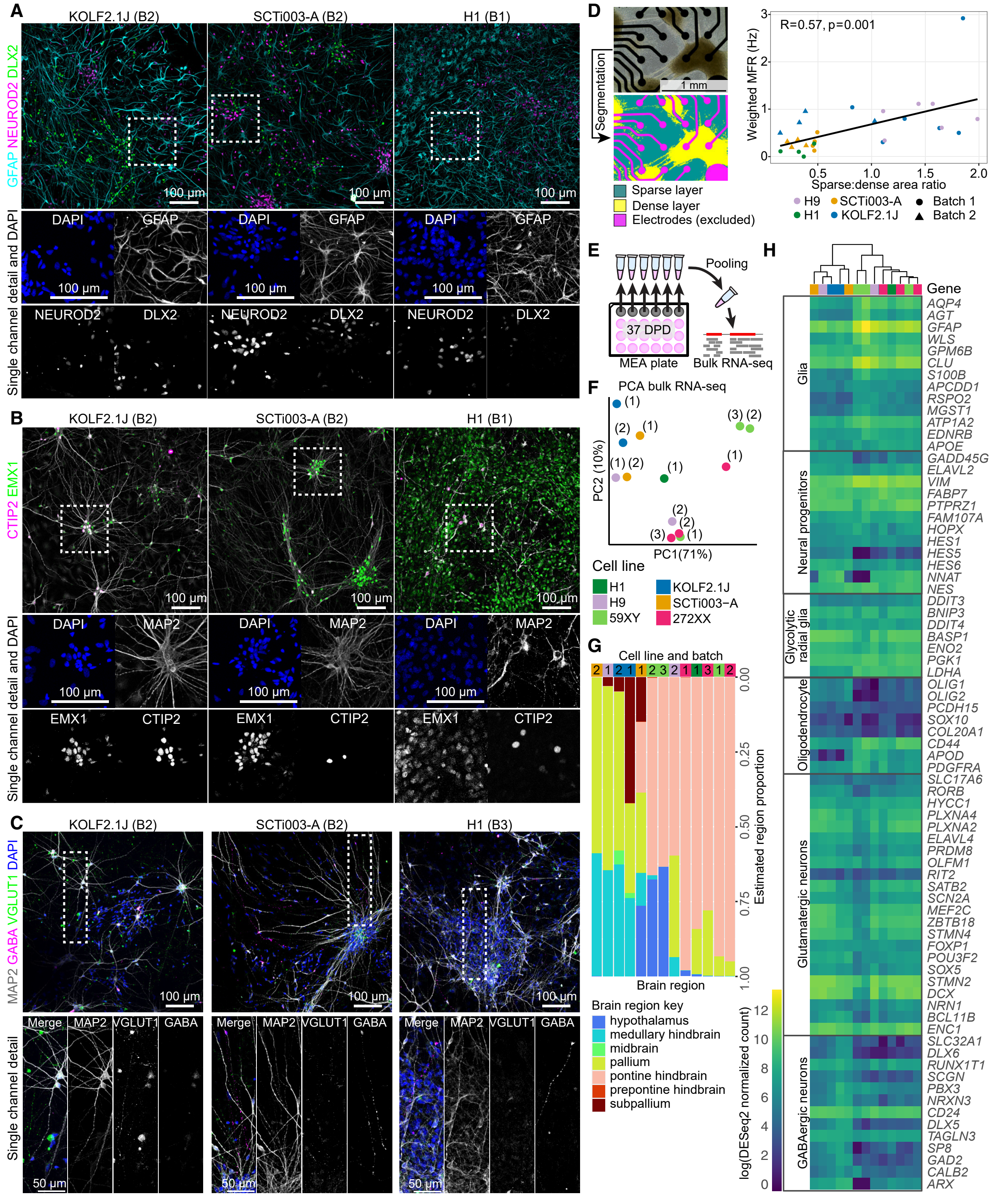

Figure 5. Dissociated organoid culture morphology, cell type, and regional identity are potential contributors to variation in MEA parameters between batches

(A–C) Immunocytochemistry for excitatory and inhibitory neuronal markers and the glial marker GFAP in dissociated organoids grown in parallel to MEA experiments at 37 DPD. Brightness and contrast adjusted for clarity. Cells from a highly active batch (KOLF2.1J batch 2, left), medium active batch (SCTi003-A

(legend continued on next page)

protocols have shown less consistent cell type and region representation and batch effects.[47,72] Consistent with unguided protocols, we found batch and cell line effects extend to development of electrophysiological activity. We would therefore expect dissociated cultures from guided organoids to show a more consistent electrophysiological phenotype, but this remains to be tested. In our assessment of cell-type composition, we showed that dissociated organoids contain a mixture of inhibitory and excitatory neurons. In iPSC co-cultures, synaptic integration of GABAergic neurons with glutamatergic neurons reduces firing and network bursting, with higher GABAergic neuron to glutamatergic neuron ratio inversely correlating with network bursting and firing rates[73]; variability in spontaneous activity could thus be determined by GABAergic neurons. Consistent with this, previous whole and dissociated organoid studies have reported that the ratio of glutamatergic:GABAergic neurons can affect network activity parameters.[41,43] Similarly, astrocytes are critical for neuronal plasticity, synapse function, and synapse formation.[74] Lower numbers of astrocytes in neuron and astrocyte co-cultures on an MEA have been shown to enhance development of network activity.[70] In line with this, we observed an apparent difference in the number of GFAP-positive cells in the H1 dissociated culture with minimal spontaneous activity. Variation in organoid differentiation is affected by starting iPSC gene expression and quality.[75] While ideally controlled, we suggest that simply assessing or directly manipulating cell-type composition in future experiments, including the incorporation of microglia,[76] could be exploited to better understand cell-type contributions to electrophysiological phenotypes.

Previous studies have demonstrated changes in organoid network activity following electrical stimulation and drug treatments,[41,69] although most only looked at acute changes in network activity of less than one hour. In dopaminergic and cortical neurons co-cultured with astrocytes, Pré et al.[49] observed increased firing rate and network burst frequency following chLTP that lasted up to 72 h and varied in magnitude between experiments. We observed similar timing and variability in the chLTP response, with increased firing and network bursting, and a more consistent response for less variable parameters, such as ISI coefficient of variation and IBI. With careful consideration of parameter selection,[18] chLTP can thus be extended for use in more complex cultures and cerebral organoids. The absence of an E2 effect on spontaneous activity may reflect that functional changes reported previously were observed either at higher, non-physiological concentrations in human iPSC neurons[68,77] or using electrical stimulation and patch camping in rodents,[78–80] which may detect synaptic changes not apparent in spontaneous activity measures.

Our method of dissociated unguided organoid cultures combines the cellular diversity and initial developmental patterning of organoids but improves the accessibility, scalability, and throughput of traditional 3D models by leveraging the advantages of 2D systems. We showed here that even unguided cerebral organoids dissociated to 2D cultures consistently develop neural networks over time, and show long-term plastic responses to stimuli, with some variation that we attribute to differences in cell composition and ratio in the cultures.

Limitations of the study

Organoid dissociation involves the loss of 3D organization and structure, which potentially disrupts any neuronal networks that may have formed before dissociation. The MEA setup used in this study does not capture activity at a single-cell resolution, and high-density MEAs may allow for more accurate network analysis. While we demonstrate the importance of batch and cell line effects, a greater number of cell lines is required to infer any meaningful impacts of cell line sex and differences between embryonic and induced pluripotent stem cell lines.

RESOURCE AVAILABILITY

Lead contact

Further information and requests for resources and reagents should be directed to and will be fulfilled by the lead contact, Deepak P. Srivastava (deepak.srivastava@kcl.ac.uk).

Materials availability

This study did not generate new unique reagents.

Data and code availability

- Bulk RNA-seq data have been deposited at GEO: GSE292624. Raw MEA data and well average data for all parameters have been deposited at King's Open Research Data System (KORDS) at https://doi.org/10.18742/c.8097928. These are all publicly available as of the date of publication. Microscopy data reported in this paper will be shared by the lead contact upon request.
- This paper does not report original code.
- Any additional information required to reanalyze the data reported in this paper is available from the lead contact upon request.

batch 2, middle), and less active batch (H1 batch 1, right). Regions indicated by the box (white dotted line) are shown enlarged below each main image, with DAPI (blue) and individual channels shown in grayscale. Immunostaining for GFAP, NEUROD2, and DLX2 (A); MAP2, EMX1, and CTIP2 (B); and VGLUT1 and GABA (C).
(D) Analysis of cell clustering in dissociated cultures and correlation to weighted MFR. The colored images show segmentation into sparse regions (blue), cluster regions (yellow), and electrodes (magenta). The brightfield image (top left) is a well from KOLF2.1J batch 1, at 37 DPD. Dot plot of correlation of cluster ratio (ratio of sparse area to dense area) at 37 DPD (*x* axis) compared to weighted mean firing rate (Hz) at 37 DPD. Points represent wells. Point shape indicates batch, and color indicates cell line. Pearson's correlation test, $N = 28$ wells. The black line represents the linear regression best fit.
(E) Schematic of pooling RNA from all wells within a cell line and batch from an MEA plate for bulk RNA-seq.
(F) PCA plot of the 500 most variable genes in the RNA-seq data. The first and second principal components are plotted, where each point represents one sample of pooled wells, point color indicates the cell line, and brackets indicate the batch number.
(G) Estimated representation of brain structures in dissociated organoids based on deconvolution of bulk RNA-seq data using Allen Brain Institute data. Columns represent samples, and colors represent the proportion of each brain region. Column annotations indicate the sample cell line (color) and batch (number).
(H) Heatmap of expression of genes (row annotations, right) from cell-type signatures (row annotations, left). Genes and categories adapted from Hendriks et al.[63] The color scale represents the log(DESeq2 normalized counts), with yellow representing the highest expression. Column annotations indicate the sample cell line (color) and batch (brackets). The dendrogram (top) represents the hierarchical clustering of the samples.

CellPress
OPEN ACCESS

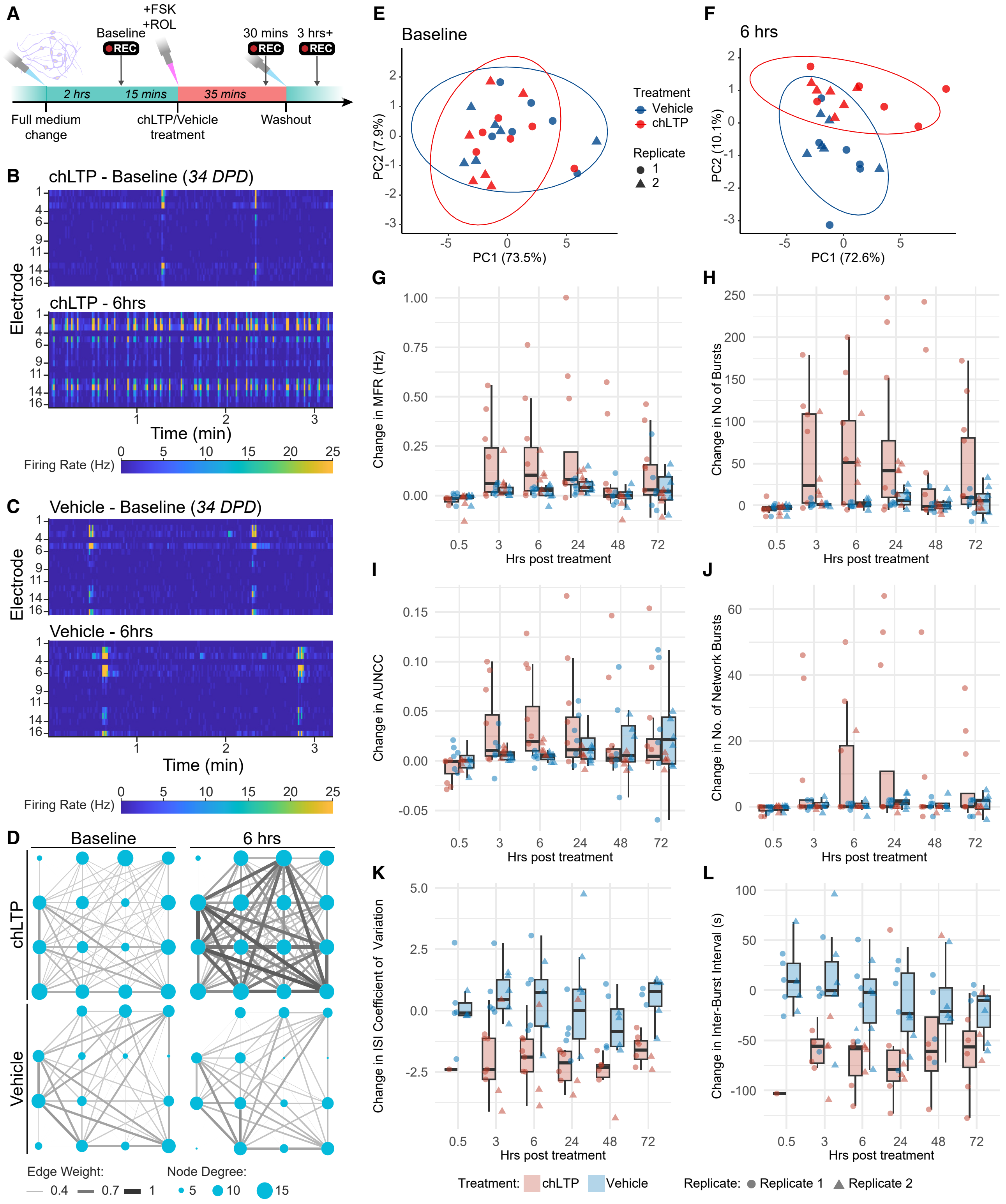

Figure 6. Dissociated organoids respond to a chemical plasticity stimulus

(A) Schematic of chLTP treatment and data collection timeline.

(B and C) Raster plots of firing rate at 34 DPD for two representative wells at baseline and 6 h following chLTP (C) or vehicle (D). The raster plots correspond to the two wells and time points shown in (B). The raster plots show MFR in hertz (Hz, color bar) in 1-s bins for each electrode (rows) over 3 min scaled to the range of

(legend continued on next page)

MFR in the entire dataset. (D) Scaled network plots showing functional connectivity at 34 DPD for two representative wells at baseline (left) and 6 h following chLTP (top right) or vehicle exposure (bottom right). The nodes (circles) represent the neuronal activity observed from neuron(s) at each electrode in the spatial arrangement of the MEA. The node degree (size of circle) represents the number of functional connections with other nodes. The edges (lines) represent significant functional connections between nodes, and the edge weight (line thickness) represents the strength of connectivity.

(E and F) PCA of 13 MEA parameters measured at baseline (E) and 6 h following chLTP (F). PCA plots of the first and second principal components. Each point represents one MEA well, colored by treatment. Point shape indicates the replicate. The data ellipses represent the 95% confidence interval, expected to enclose 95% of bivariate-normal t distributed data for each treatment condition.

(G–L) Boxplots of difference relative to baseline in MEA parameters after chLTP or vehicle treatment. Boxplots show the min, max, median, and IQR of the change in MFR (G), number of bursts/5 min (H), AUNCC (I), NBN/5 min (J), ISI coefficient of variation (K), and inter-burst interval (L) per well, at six different time points (0.5, 3, 6, 24, 48, and 72 h post-treatment). Points represent the mean of one well across 5 min of recording. Point color indicates treatment (vehicle, blue; chLTP, red), and shape indicates the replicate. In (K and L), only wells with bursts present are shown. Measured in KOLF2.1J line dissociated organoids, with treatment starting at 34 DPD (replicate one) and 29 DPD (replicate 2).

CONSORTIA

The consortium members are as follows: Simon Baron-Cohen, Deep Adhya, Deepak P Srivastava, Lucia Dutan-Polit, Adam Pavlinek, Roland Nagy, Madeline A Lancaster, Jose Gonzalez-Martinez, Jonathan Mill, Alice Franklin, Rosie Bamford, Daniel H Geschwind, Ramin Ali Marandi Ghoddousi, Carrie Allison, Varun Warrier, Alex Tsompanidis, Alexander EP Heazell, Matthew E Hurles, Hilary C Martin, Mahmoud Mousa, David H Rowitch, Kathy K Niakan, Graham J Burton, Fateneh Ghafari, Tal Biron-Shental, Lidia V Gabis, Rosie Holt, Joanna Davis, Genie Gu, Yira Zhang, Niran Okewole, and Omar Al-Rubaie.

ACKNOWLEDGMENTS

The authors acknowledge funding support from UK Medical Research Council, (grant nos. MR/L021064/1 [D.P.S.], MR/Y012968/1 [A.C.V. and D.P.S.], MR/X004112/1 [D.P.S.], MR/Y008170/1 [D.P.S.], and MR/Y012968/1 [A.C.V. and D.P.S.]) and from The Simons Foundation Autism Research Initiative (SFARI). D.P.S. is also a recipient of an Independent Researcher Award from the Brain and Behavior Foundation (grant no. 25957). A.P. is in receipt of the MRC Institute for Translational Neurodevelopment (ITND) Ph.D. studentship, as part of the MRC Centre for Neurodevelopmental Disorders, Medical Research Council (MR/P502108/1). A.P. and D.P.S. also acknowledge funding from Psychiatry Research Trust. The authors acknowledge use of King's Computational Research, Engineering and Technology Environment (CREATE). The authors thank George Chenell of the Wohl Cellular Imaging Center (King's College London) for technical support, Niamh O'Brien for advice on organoid dissociation methods, and Iva Kelava for training in cerebral organoid culture.

AUTHOR CONTRIBUTIONS

Conceptualization, A.P., A.C.V., and D.P.S.; methodology, A.P., A.C.V., and D.P.S.; investigation, A.P., S.G., K.O., L.D.P., and R.N.; writing—original draft, A.P.; visualization, A.P., writing—review & editing, M.A.L., A.C.V., and D.P.S.; funding acquisition, M.A.L., A.C.V., and D.P.S.; resources, M.L.; supervision, M.A.L., A.C.V., and D.P.S.

DECLARATION OF INTERESTS

The authors declare no competing interests.

STAR★METHODS

Detailed methods are provided in the online version of this paper and include the following:

- KEY RESOURCES TABLE
- EXPERIMENTAL MODEL AND STUDY PARTICIPANT DETAILS
  - Cell line information
- METHOD DETAILS
  - Generation of unguided cerebral organoids
  - Dissociation of cerebral organoids for culture on MEA
  - Dissociated organoid plating and MEA culture
  - MEA recording
  - Treatment experiments
  - Immunocytochemistry
  - RNA-seq
- QUANTIFICATION AND STATISTICAL ANALYSIS
  - MEA data analysis
  - Statistical analysis
  - Dimensionality reduction analysis
  - Analysis of dissociated organoid culture morphology
  - Bulk RNA-seq analysis
  - Analysis of estimated organoid regional identity

SUPPLEMENTAL INFORMATION

Supplemental information can be found online at https://doi.org/10.1016/j.crmeth.2026.101371.

Received: April 8, 2025
Revised: December 12, 2025
Accepted: February 26, 2026
Published: March 27, 2026

REFERENCES

1. Power, J.D., Fair, D.A., Schlaggar, B.L., and Petersen, S.E. (2010). The development of human functional brain networks. Neuron *67*, 735–748. https://doi.org/10.1016/j.neuron.2010.08.017.
2. Wong, H.H.W., Chou, C.Y.C., Watt, A.J., and Sjöström, P.J. (2023). Comparing mouse and human brains. eLife *12*, e90017. https://doi.org/10.7554/eLife.90017.
3. Mansvelder, H.D., Verhoog, M.B., and Goriounova, N.A. (2019). Synaptic plasticity in human cortical circuits: cellular mechanisms of learning and memory in the human brain? Curr. Opin. Neurobiol. *54*, 186–193. https://doi.org/10.1016/j.conb.2018.06.013.
4. Beauchamp, A., Yee, Y., Darwin, B.C., Raznahan, A., Mars, R.B., and Lerch, J.P. (2022). Whole-brain comparison of rodent and human brains using spatial transcriptomics. eLife *11*, e79418. https://doi.org/10.7554/eLife.79418.
5. Berg, J., Sorensen, S.A., Ting, J.T., Miller, J.A., Chartrand, T., Buchin, A., Bakken, T.E., Budzillo, A., Dee, N., Ding, S.-L., et al. (2021). Human neocortical expansion involves glutamatergic neuron diversification. Nature *598*, 151–158. https://doi.org/10.1038/s41586-021-03813-8.
6. Florio, M., and Huttner, W.B. (2014). Neural progenitors, neurogenesis and the evolution of the neocortex. Development *141*, 2182–2194. https://doi.org/10.1242/dev.090571.
7. Bakken, T.E., Jorstad, N.L., Hu, Q., Lake, B.B., Tian, W., Kalmbach, B.E., Crow, M., Hodge, R.D., Krienen, F.M., Sorensen, S.A., et al. (2021).

Comparative cellular analysis of motor cortex in human, marmoset and mouse. Nature *598*, 111–119. https://doi.org/10.1038/s41586-021-03465-8.

8. Khrameeva, E., Kurochkin, I., Han, D., Guijarro, P., Kanton, S., Santel, M., Qian, Z., Rong, S., Mazin, P., Sabirov, M., et al. (2020). Single-cell-resolution transcriptome map of human, chimpanzee, bonobo, and macaque brains. Genome Res. *30*, 776–789. https://doi.org/10.1101/gr.256958.119.
9. DeFelipe, J. (2011). The Evolution of the Brain, the Human Nature of Cortical Circuits, and Intellectual Creativity. Front. Neuroanat. *5*, 29. https://doi.org/10.3389/fnana.2011.00029.
10. Vanderhaeghen, P., and Polleux, F. (2023). Developmental mechanisms underlying the evolution of human cortical circuits. Nat. Rev. Neurosci. *24*, 213–232. https://doi.org/10.1038/s41583-023-00675-z.
11. Reillo, I., de Juan Romero, C., García-Cabezas, M.Á., and Borrell, V. (2011). A role for intermediate radial glia in the tangential expansion of the mammalian cerebral cortex. Cereb. Cortex *21*, 1674–1694. https://doi.org/10.1093/cercor/bhq238.
12. Wallace, J.L., and Pollen, A.A. (2024). Human neuronal maturation comes of age: cellular mechanisms and species differences. Nat. Rev. Neurosci. *25*, 7–29. https://doi.org/10.1038/s41583-023-00760-3.
13. Wang, L., Pang, K., Zhou, L., Cebrián-Silla, A., González-Granero, S., Wang, S., Bi, Q., White, M.L., Ho, B., Li, J., et al. (2023). A cross-species proteomic map reveals neoteny of human synapse development. Nature *622*, 112–119. https://doi.org/10.1038/s41586-023-06542-2.
14. Espuny-Camacho, I., Michelsen, K.A., Gall, D., Linaro, D., Hasche, A., Bonnefont, J., Bali, C., Orduz, D., Bilheu, A., Herpoel, A., et al. (2013). Pyramidal neurons derived from human pluripotent stem cells integrate efficiently into mouse brain circuits in vivo. Neuron *77*, 440–456. https://doi.org/10.1016/j.neuron.2012.12.011.
15. Linaro, D., Vermaercke, B., Iwata, R., Ramaswamy, A., Libé-Philippot, B., Boubakar, L., Davis, B.A., Wierda, K., Davie, K., Poovathingal, S., et al. (2019). Xenotransplanted Human Cortical Neurons Reveal Species-Specific Development and Functional Integration into Mouse Visual Circuits. Neuron *104*, 972–986.e6. https://doi.org/10.1016/j.neuron.2019.10.002.
16. Petanjek, Z., Judaš, M., Šimic, G., Rasin, M.R., Uylings, H.B.M., Rakic, P., and Kostovic, I. (2011). Extraordinary neoteny of synaptic spines in the human prefrontal cortex. Proc. Natl. Acad. Sci. USA *108*, 13281–13286. https://doi.org/10.1073/pnas.1105108108.
17. Ronchi, S., Buccino, A.P., Prack, G., Kumar, S.S., Schröter, M., Fiscella, M., and Hierlemann, A. (2021). Electrophysiological Phenotype Characterization of Human iPSC-Derived Neuronal Cell Lines by Means of High-Density Microelectrode Arrays. Adv. Biol. *5*, 2000223. https://doi.org/10.1002/adbi.202000223.
18. Mossink, B., Verboven, A.H.A., van Hugte, E.J.H., Gunnewiek, T.M.K., Parodi, G., Linda, K., Schoenmaker, C., Kleefstra, T., Kozicz, T., van Bokhoven, H., et al. (2021). Human neuronal networks on micro-electrode arrays are a highly robust tool to study disease-specific genotype-phenotype correlations in vitro. bioRxiv. https://doi.org/10.1101/2021.01.20.427439.
19. Odawara, A., Katoh, H., Matsuda, N., and Suzuki, I. (2016). Physiological maturation and drug responses of human induced pluripotent stem cell-derived cortical neuronal networks in long-term culture. Sci. Rep. *6*, 26181. https://doi.org/10.1038/srep26181.
20. Rosa, F., Dhingra, A., Uysal, B., Mendis, G.D.C., Loeffler, H., Elsen, G., Mueller, S., Schwarz, N., Castillo-Lizardo, M., Cuddy, C., et al. (2020). In Vitro Differentiated Human Stem Cell-Derived Neurons Reproduce Synaptic Synchronicity Arising during Neurodevelopment. Stem Cell Rep. *15*, 22–37. https://doi.org/10.1016/j.stemcr.2020.05.015.
21. Izsak, J., Seth, H., Andersson, M., Vizlin-Hodzic, D., Theiss, S., Hanse, E., Ågren, H., Funa, K., and Illes, S. (2019). Robust Generation of Person-Specific, Synchronously Active Neuronal Networks Using Purely Isogenic Human iPSC-3D Neural Aggregate Cultures. Front. Neurosci. *13*, 351. https://doi.org/10.3389/fnins.2019.00351.
22. Bakkum, D.J., Frey, U., Radivojevic, M., Russell, T.L., Müller, J., Fiscella, M., Takahashi, H., and Hierlemann, A. (2013). Tracking axonal action potential propagation on a high-density microelectrode array across hundreds of sites. Nat. Commun. *4*, 2181. https://doi.org/10.1038/ncomms3181.
23. Suresh, J., Radojicic, M., Pesce, L.L., Bhansali, A., Wang, J., Tryba, A.K., Marks, J.D., and van Drongelen, W. (2016). Network burst activity in hippocampal neuronal cultures: the role of synaptic and intrinsic currents. J. Neurophysiol. *115*, 3073–3089. https://doi.org/10.1152/jn.00995.2015.
24. Sit, T.P.H., Feord, R.C., Dunn, A.W.E., Chabros, J., Oluigbo, D., Smith, H.H., Burn, L., Chang, E., Boschi, A., Yuan, Y., et al. (2024). MEA-NAP: A flexible network analysis pipeline for neuronal 2D and 3D organoid multielectrode recordings. Cell Rep. Methods *4*, 100901. https://doi.org/10.1016/j.crmeth.2024.100901.
25. Uhlhaas, P.J., Roux, F., Rodriguez, E., Rotarska-Jagiela, A., and Singer, W. (2010). Neural synchrony and the development of cortical networks. Trends Cogn. Sci. *14*, 72–80. https://doi.org/10.1016/j.tics.2009.12.002.
26. Pan, Y., and Monje, M. (2020). Activity Shapes Neural Circuit Form and Function: A Historical Perspective. J. Neurosci. *40*, 944–954. https://doi.org/10.1523/JNEUROSCI.0740-19.2019.
27. Ross, P.J., Mok, R.S.F., Smith, B.S., Rodrigues, D.C., Mufteev, M., Scherer, S.W., and Ellis, J. (2020). Modeling neuronal consequences of autism-associated gene regulatory variants with human induced pluripotent stem cells. Mol. Autism *11*, 33. https://doi.org/10.1186/s13229-020-00333-6.
28. Deneault, E., Faheem, M., White, S.H., Rodrigues, D.C., Sun, S., Wei, W., Piekna, A., Thompson, T., Howe, J.L., Chalil, L., et al. (2019). CNTN5-/+or EHMT2-/+human iPSC-derived neurons from individuals with autism develop hyperactive neuronal networks. eLife *8*, e40092. https://doi.org/10.7554/eLife.40092.
29. Deneault, E., White, S.H., Rodrigues, D.C., Ross, P.J., Faheem, M., Zaslavsky, K., Wang, Z., Alexandrova, R., Pellecchia, G., Wei, W., et al. (2018). Complete Disruption of Autism-Susceptibility Genes by Gene Editing Predominantly Reduces Functional Connectivity of Isogenic Human Neurons. Stem Cell Rep. *11*, 1211–1225. https://doi.org/10.1016/j.stemcr.2018.10.003.
30. Kelley, K.W., and Pașca, S.P. (2022). Human brain organogenesis: Toward a cellular understanding of development and disease. Cell *185*, 42–61. https://doi.org/10.1016/j.cell.2021.10.003.
31. Velasco, S., Kedaigle, A.J., Simmons, S.K., Nash, A., Rocha, M., Quadrato, G., Paulsen, B., Nguyen, L., Adiconis, X., Regev, A., et al. (2019). Individual brain organoids reproducibly form cell diversity of the human cerebral cortex. Nature *570*, 523–527. https://doi.org/10.1038/s41586-019-1289-x.
32. Camp, J.G., Badsha, F., Florio, M., Kanton, S., Gerber, T., Wilsch-Bräuninger, M., Lewitus, E., Sykes, A., Hevers, W., Lancaster, M., et al. (2015). Human cerebral organoids recapitulate gene expression programs of fetal neocortex development. Proc. Natl. Acad. Sci. USA *112*, 15672–15677. https://doi.org/10.1073/pnas.1520760112.
33. Tanaka, Y., Cakir, B., Xiang, Y., Sullivan, G.J., and Park, I.-H. (2020). Synthetic Analyses of Single-Cell Transcriptomes from Multiple Brain Organoids and Fetal Brain. Cell Rep. *30*, 1682–1689.e3. https://doi.org/10.1016/j.celrep.2020.01.038.
34. Cheroni, C., Trattaro, S., Caporale, N., López-Tobón, A., Tenderini, E., Sebastiani, S., Troglio, F., Gabriele, M., Bressan, R.B., Pollard, S.M., et al. (2022). Benchmarking brain organoid recapitulation of fetal corticogenesis. Transl. Psychiatry *12*, 520. https://doi.org/10.1038/s41398-022-02279-0.
35. Amiri, A., Coppola, G., Scuderi, S., Wu, F., Roychowdhury, T., Liu, F., Pochareddy, S., Shin, Y., Safi, A., Song, L., et al. (2018). Transcriptome and epigenome landscape of human cortical development modeled in organoids. Science *362*, eaat6720. https://doi.org/10.1126/science.aat6720.

36. Ziffra, R.S., Kim, C.N., Ross, J.M., Wilfert, A., Turner, T.N., Haeussler, M., Casella, A.M., Przytycki, P.F., Keough, K.C., Shin, D., et al. (2021). Single-cell epigenomics reveals mechanisms of human cortical development. Nature *598*, 205–213. https://doi.org/10.1038/s41586-021-03209-8.

37. Chiaradia, I., Imaz-Rosshandler, I., Nilges, B.S., Boulanger, J., Pellegrini, L., Das, R., Kashikar, N.D., and Lancaster, M.A. (2023). Tissue morphology influences the temporal program of human brain organoid development. Cell Stem Cell *30*, 1351–1367.e10. https://doi.org/10.1016/j.stem.2023.09.003.

38. Paşca, S.P., Arlotta, P., Bateup, H.S., Camp, J.G., Cappello, S., Gage, F.H., Knoblich, J.A., Kriegstein, A.R., Lancaster, M.A., Ming, G.-L., et al. (2022). A nomenclature consensus for nervous system organoids and assembloids. Nature *609*, 907–910. https://doi.org/10.1038/s41586-022-05219-6.

39. Del Dosso, A., Urenda, J.-P., Nguyen, T., and Quadrato, G. (2020). Upgrading the Physiological Relevance of Human Brain Organoids. Neuron *107*, 1014–1028. https://doi.org/10.1016/j.neuron.2020.08.029.

40. Xiang, Y., Tanaka, Y., Patterson, B., Kang, Y.-J., Govindaiah, G., Roselaar, N., Cakir, B., Kim, K.-Y., Lombroso, A.P., Hwang, S.-M., et al. (2017). Fusion of Regionally Specified hPSC-Derived Organoids Models Human Brain Development and Interneuron Migration. Cell Stem Cell *21*, 383–398.e7. https://doi.org/10.1016/j.stem.2017.07.007.

41. Sakaguchi, H., Ozaki, Y., Ashida, T., Matsubara, T., Oishi, N., Kihara, S., and Takahashi, J. (2019). Self-Organized Synchronous Calcium Transients in a Cultured Human Neural Network Derived from Cerebral Organoids. Stem Cell Rep. *13*, 458–473. https://doi.org/10.1016/j.stemcr.2019.05.029.

42. Trujillo, C.A., Gao, R., Negraes, P.D., Gu, J., Buchanan, J., Preissl, S., Wang, A., Wu, W., Haddad, G.G., Chaim, I.A., et al. (2019). Complex Oscillatory Waves Emerging from Cortical Organoids Model Early Human Brain Network Development. Cell Stem Cell *25*, 558–569.e7. https://doi.org/10.1016/j.stem.2019.08.002.

43. Fair, S.R., Julian, D., Hartlaub, A.M., Pusuluri, S.T., Malik, G., Summerfied, T.L., Zhao, G., Hester, A.B., Ackerman, W.E., Hollingsworth, E.W., et al. (2020). Electrophysiological Maturation of Cerebral Organoids Correlates with Dynamic Morphological and Cellular Development. Stem Cell Rep. *15*, 855–868. https://doi.org/10.1016/j.stemcr.2020.08.017.

44. Giandomenico, S.L., Mierau, S.B., Gibbons, G.M., Wenger, L.M.D., Masullo, L., Sit, T., Sutcliffe, M., Boulanger, J., Tripodi, M., Derivery, E., et al. (2019). Cerebral organoids at the air–liquid interface generate diverse nerve tracts with functional output. Nat. Neurosci. *22*, 669–679. https://doi.org/10.1038/s41593-019-0350-2.

45. Yan, Y., Arzua, T., Logan, S., and Bai, X. (2022). Isolation and Culture of Human-Induced Pluripotent Stem Cell-Derived Cerebral Organoid Cells. Methods Mol. Biol. *2454*, 483–494. https://doi.org/10.1007/7651_2020_328.

46. Park, S.B., Koh, B., Kwon, H.S., Kim, Y.E., Kim, S.S., Cho, S.-H., Kim, T.-Y., Bae, M.A., Kang, D., Kim, C.H., and Kim, K.Y. (2023). Quantitative and Qualitative Analysis of Neurotransmitter and Neurosteroid Production in Cerebral Organoids during Differentiation. ACS Chem. Neurosci. *14*, 3761–3771. https://doi.org/10.1021/acschemneuro.3c00246.

47. Naas, J., Balmaña, M., Holcik, L., Novatchkova, M., Dobnikar, L., Krausgruber, T., Ladstätter, S., Bock, C., von Haeseler, A., Esk, C., and Knoblich, J.A. (2025). Quantitative profiling of human brain organoid cell diversity across four protocols and multiple cell lines. Cell Rep. *44*, 116168. https://doi.org/10.1016/j.celrep.2025.116168.

48. Sharf, T., van der Molen, T., Glasauer, S.M.K., Guzman, E., Buccino, A.P., Luna, G., Cheng, Z., Audouard, M., Ranasinghe, K.G., Kudo, K., et al. (2022). Functional neuronal circuitry and oscillatory dynamics in human brain organoids. Nat. Commun. *13*, 4403. https://doi.org/10.1038/s41467-022-32115-4.

49. Pré, D., Wooten, A.T., Biesmans, S., Hinckley, S., Zhou, H., Sherman, S.P., Kakad, P., Gearhart, J., and Bang, A.G. (2022). Development of a platform to investigate long-term potentiation in human iPSC-derived neuronal networks. Stem Cell Rep. *17*, 2141–2155. https://doi.org/10.1016/j.stemcr.2022.07.012.

50. Bi, R., Broutman, G., Foy, M.R., Thompson, R.F., and Baudry, M. (2000). The tyrosine kinase and mitogen-activated protein kinase pathways mediate multiple effects of estrogen in hippocampus. Proc. Natl. Acad. Sci. USA *97*, 3602–3607. https://doi.org/10.1073/pnas.97.7.3602.

51. Clements, L., and Harvey, J. (2020). Activation of oestrogen receptor α induces a novel form of LTP at hippocampal temporoammonic-CA1 synapses. Br. J. Pharmacol. *177*, 642–655. https://doi.org/10.1111/bph.14880.

52. Corina, E.A., Bogdan, A.M., Elize, D.H., Piet, K., Frank, H.D.J., Andrée, K., Chris, I.D.Z., and Marcel, T.G.D.J. (2007). Estradiol Improves Cerebellar Memory Formation by Activating Estrogen Receptor β. J. Neurosci. *27*, 10832. https://doi.org/10.1523/JNEUROSCI.2588-07.2007.

53. Scarduzio, M., Panichi, R., Pettorossi, V.E., and Grassi, S. (2013). Synaptic Long-Term Potentiation and Depression in the Rat Medial Vestibular Nuclei Depend on Neural Activation of Estrogenic and Androgenic Signals. PLoS One *8*, e80792. https://doi.org/10.1371/journal.pone.0080792.

54. Foy, M.R., Xu, J., Xie, X., Brinton, R.D., Thompson, R.F., and Berger, T.W. (1999). 17β-Estradiol Enhances NMDA Receptor-Mediated EPSPs and Long-Term Potentiation. J. Neurophysiol. *81*, 925–929. https://doi.org/10.1152/jn.1999.81.2.925.

55. Grassi, S., Frondaroli, A., Scarduzio, M., Dutia, M.B., Dieni, C., and Pettorossi, V.E. (2010). Effects of 17B-estradiol on glutamate synaptic transmission and neuronal excitability in the rat medial vestibular nuclei. Neuroscience *165*, 1100–1114. https://doi.org/10.1016/j.neuroscience.2009.11.039.

56. Hasegawa, Y., Hojo, Y., Kojima, H., Ikeda, M., Hotta, K., Sato, R., Ooishi, Y., Yoshiya, M., Chung, B.-C., Yamazaki, T., and Kawato, S. (2015). Estradiol rapidly modulates synaptic plasticity of hippocampal neurons: Involvement of kinase networks. Brain Res. *1621*, 147–161. https://doi.org/10.1016/j.brainres.2014.12.056.

57. Lancaster, M.A., Corsini, N.S., Wolfinger, S., Gustafson, E.H., Phillips, A.W., Burkard, T.R., Otani, T., Livesey, F.J., and Knoblich, J.A. (2017). Guided self-organization and cortical plate formation in human brain organoids. Nat. Biotechnol. *35*, 659–666. https://doi.org/10.1038/nbt.3906.

58. Lancaster, M.A., Renner, M., Martin, C.-A., Wenzel, D., Bicknell, L.S., Hurles, M.E., Homfray, T., Penninger, J.M., Jackson, A.P., and Knoblich, J.A. (2013). Cerebral organoids model human brain development and microcephaly. Nature *501*, 373–379. https://doi.org/10.1038/nature12517.

59. Renner, M., Lancaster, M.A., Bian, S., Choi, H., Ku, T., Peer, A., Chung, K., and Knoblich, J.A. (2017). Self-organized developmental patterning and differentiation in cerebral organoids. EMBO J. *36*, 1316–1329. https://doi.org/10.15252/embj.201694700.

60. Paşca, S.P., Arlotta, P., Bateup, H.S., Camp, J.G., Cappello, S., Gage, F.H., Knoblich, J.A., Kriegstein, A.R., Lancaster, M.A., Ming, G.-L., et al. (2025). A framework for neural organoids, assembloids and transplantation studies. Nature *639*, 315–320. https://doi.org/10.1038/s41586-024-08487-6.

61. Halliday, D.M., Rosenberg, J.R., Breeze, P., and Conway, B.A. (2006). Neural spike train synchronization indices: definitions, interpretations, and applications. IEEE Trans. Biomed. Eng. *53*, 1056–1066. https://doi.org/10.1109/tbme.2006.873392.

62. Pantazis, C.B., Yang, A., Lara, E., McDonough, J.A., Blauwendraat, C., Peng, L., Oguro, H., Kanaujiya, J., Zou, J., Sebesta, D., et al. (2022). A reference human induced pluripotent stem cell line for large-scale collaborative studies. Cell Stem Cell *29*, 1685–1702.e22. https://doi.org/10.1016/j.stem.2022.11.004.

63. Hendriks, D., Pagliaro, A., Andreatta, F., Ma, Z., van Giessen, J., Massalini, S., López-Iglesias, C., van Son, G.J.F., DeMartino, J., Damen, J.M.A., et al. (2024). Human fetal brain self-organizes into long-term expanding organoids. Cell *187*, 712–732.e38. https://doi.org/10.1016/j.cell.2023.12.012.

CellPress
OPEN ACCESS

64. Fleck, J.S., Sanchís-Calleja, F., He, Z., Santel, M., Boyle, M.J., Camp, J.G., and Treutlein, B. (2021). Resolving organoid brain region identities by mapping single-cell genomic data to reference atlases. Cell Stem Cell *28*, 1148–1159.e1148. https://doi.org/10.1016/j.stem.2021.02.015.

65. Michael Deans, P.J., Shum, C., Perfect, L., Conforti, M., Duarte, R., Bhattacharyya, S., Brandon, N., Price, J., and Srivastava, D. (2018). O10.7. INVESTIGATING THE MECHANISMS UNDERLYING THE BENEFICIAL EFFECTS OF ESTROGENS IN SCHIZOPHRENIA. Schizophr. Bull. *44*, S105. https://doi.org/10.1093/schbul/sby015.259.

66. Shum, C., Macedo, S.C., Warre-Cornish, K., Cocks, G., Price, J., and Srivastava, D.P. (2015). Utilizing induced pluripotent stem cells (iPSCs) to understand the actions of estrogens in human neurons. Horm. Behav. *74*, 228–242. https://doi.org/10.1016/j.yhbeh.2015.06.014.

67. Acharya, K.D., Nettles, S.A., Lichti, C.F., Warre-Cornish, K., Dutan Polit, L., Srivastava, D.P., Denner, L., and Tetel, M.J. (2020). Dopamine-induced interactions of female mouse hypothalamic proteins with progestin receptor-A in the absence of hormone. J. Neuroendocrinol. *32*, e12904. https://doi.org/10.1111/jne.12904.

68. Supakul, S., Oyama, C., Hatakeyama, Y., Maeda, S., and Okano, H. (2024). Estradiol enhanced neuronal plasticity and ameliorated astrogliosis in human iPSC-derived neural models. Regen. Ther. *25*, 250–263. https://doi.org/10.1016/j.reth.2023.12.018.

69. Zafeiriou, M.-P., Bao, G., Hudson, J., Halder, R., Blenkle, A., Schreiber, M.-K., Fischer, A., Schild, D., and Zimmermann, W.-H. (2020). Developmental GABA polarity switch and neuronal plasticity in Bioengineered Neuronal Organoids. Nat. Commun. *11*, 3791. https://doi.org/10.1038/s41467-020-17521-w.

70. Hörberg, C.-J., Englund Johansson, U., Johansson, F., and O'Carroll, D. (2022). Spontaneous Cell Cluster Formation in Human iPSC-Derived Neuronal Spheroid Networks Influences Network Activity. eNeuro *9*, ENEURO.0143-0122.2022. https://doi.org/10.1523/ENEURO.0143-22.2022.

71. Yoon, S.-J., Elahi, L.S., Pașca, A.M., Marton, R.M., Gordon, A., Revah, O., Miura, Y., Walczak, E.M., Holdgate, G.M., Fan, H.C., et al. (2019). Reliability of human cortical organoid generation. Nat. Methods *16*, 75–78. https://doi.org/10.1038/s41592-018-0255-0.

72. Quadrato, G., Nguyen, T., Macosko, E.Z., Sherwood, J.L., Min Yang, S., Berger, D.R., Maria, N., Scholvin, J., Goldman, M., Kinney, J.P., et al. (2017). Cell diversity and network dynamics in photosensitive human brain organoids. Nature *545*, 48–53. https://doi.org/10.1038/nature22047.

73. Mossink, B., van Rhijn, J.-R., Wang, S., Linda, K., Vitale, M.R., Zöller, J.E.M., van Hugte, E.J.H., Bak, J., Verboven, A.H.A., Selten, M., et al. (2022). Cadherin-13 is a critical regulator of GABAergic modulation in human stem-cell-derived neuronal networks. Mol. Psychiatry *27*, 1–18. https://doi.org/10.1038/s41380-021-01117-x.

74. Chung, W.-S., Allen, N.J., and Eroglu, C. (2015). Astrocytes Control Synapse Formation, Function, and Elimination. Cold Spring Harb. Perspect. Biol. *7*, a020370. https://doi.org/10.1101/cshperspect.a020370.

75. Glass, M.R., Waxman, E.A., Yamashita, S., Lafferty, M., Beltran, A.A., Farah, T., Patel, N.K., Singla, R., Matoba, N., Ahmed, S., et al. (2024). Cross-site reproducibility of human cortical organoids reveals consistent cell type composition and architecture. Stem Cell Rep. *19*, 1351–1367. https://doi.org/10.1016/j.stemcr.2024.07.008.

76. Sabate-Soler, S., Nickels, S.L., Saraiva, C., Berger, E., Dubonyte, U., Barmpa, K., Lan, Y.J., Kouno, T., Jarazo, J., Robertson, G., et al. (2022). Microglia integration into human midbrain organoids leads to increased neuronal maturation and functionality. Glia *70*, 1267–1288. https://doi.org/10.1002/glia.24167.

77. Hercz, P., Ungar, L., Siklos, P., and Farquharson, R.G. (1988). Unconjugated 17β-oestradiol and destriol in maternal serum and in cord vein and artery blood at term and preterm delivery. Eur. J. Obstet. Gynecol. Reprod. Biol. *27*, 7–12. https://doi.org/10.1016/S0028-2243(88)80004-2.

78. Srivastava, D.P., Woolfrey, K.M., Jones, K.A., Shum, C.Y., Lash, L.L., Swanson, G.T., and Penzes, P. (2008). Rapid enhancement of two-step wiring plasticity by estrogen and NMDA receptor activity. Proc. Natl. Acad. Sci. USA *105*, 14650–14655. https://doi.org/10.1073/pnas.0801581105.

79. Srivastava, D.P., Woolfrey, K.M., and Penzes, P. (2013). Insights into rapid modulation of neuroplasticity by brain estrogens. Pharmacol. Rev. *65*, 1318–1350. https://doi.org/10.1124/pr.111.005272.

80. Ye, Z., Cudmore, R.H., and Linden, D.J. (2019). Estrogen-Dependent Functional Spine Dynamics in Neocortical Pyramidal Neurons of the Mouse. J. Neurosci. *39*, 4874–4888. https://doi.org/10.1523/jneurosci.2772-18.2019.

81. Ludwig, T.E., Andrews, P.W., Barbaric, I., Benvenisty, N., Bhattacharyya, A., Crook, J.M., Daheron, L.M., Draper, J.S., Healy, L.E., Huch, M., et al. (2023). ISSCR standards for the use of human stem cells in basic research. Stem Cell Rep. *18*, 1744–1752. https://doi.org/10.1016/j.stemcr.2023.08.003.

82. Giandomenico, S.L., Sutcliffe, M., and Lancaster, M.A. (2021). Generation and long-term culture of advanced cerebral organoids for studying later stages of neural development. Nat. Protoc. *16*, 579–602. https://doi.org/10.1038/s41596-020-00433-w.

83. Axion BioSystems. (2020). Neural Metric Tool User Guide, 3.1 Edition (Axion BioSystems, Inc).

84. Legéndy, C.R., and Salcman, M. (1985). Bursts and recurrences of bursts in the spike trains of spontaneously active striate cortex neurons. J. Neurophysiol. *53*, 926–939. https://doi.org/10.1152/jn.1985.53.4.926.

85. Cutts, C.S., and Eglen, S.J. (2014). Detecting pairwise correlations in spike trains: an objective comparison of methods and application to the study of retinal waves. J. Neurosci. *34*, 14288–14303. https://doi.org/10.1523/jneurosci.2767-14.2014.

86. Wickham, H. (2016). ggplot2: Elegant Graphics for Data Analysis, 2nd 2016 Edition (Springer International Publishing : Imprint: Springer).

87. Konopka, T. (2018). umap: Uniform Manifold Approximation and Projection. https://doi.org/10.32614/CRAN.package.umap.

88. Kassambara, A., and Mundt, F. (2016). factoextra: Extract and Visualize the Results of Multivariate Data Analyses. https://doi.org/10.32614/CRAN.package.factoextra.

89. Berg, S., Kutra, D., Kroeger, T., Straehle, C.N., Kausler, B.X., Haubold, C., Schiegg, M., Ales, J., Beier, T., Rudy, M., et al. (2019). ilastik: interactive machine learning for (bio)image analysis. Nat. Methods *16*, 1226–1232. https://doi.org/10.1038/s41592-019-0582-9.

90. Wingett, S.W., and Andrews, S. (2018). FastQ Screen: A tool for multi-genome mapping and quality control. F1000Res. *7*, 1338. https://doi.org/10.12688/f1000research.15931.2.

91. Dobin, A., Davis, C.A., Schlesinger, F., Drenkow, J., Zaleski, C., Jha, S., Batut, P., Chaisson, M., and Gingeras, T.R. (2013). STAR: ultrafast universal RNA-seq aligner. Bioinformatics *29*, 15–21. https://doi.org/10.1093/bioinformatics/bts635.

92. Danecek, P., Bonfield, J.K., Liddle, J., Marshall, J., Ohan, V., Pollard, M.O., Whitwham, A., Keane, T., McCarthy, S.A., Davies, R.M., and Li, H. (2021). Twelve years of SAMtools and BCFtools. GigaScience *10*, giab008. https://doi.org/10.1093/gigascience/giab008.

93. Picard Toolkit (2019). (Broad Institute). https://broadinstitute.github.io/picard/

94. Liao, Y., Smyth, G.K., and Shi, W. (2019). The R package Rsubread is easier, faster, cheaper and better for alignment and quantification of RNA sequencing reads. Nucleic Acids Res. *47*, e47. https://doi.org/10.1093/nar/gkz114.

95. Durinck, S., Spellman, P.T., Birney, E., and Huber, W. (2009). Mapping identifiers for the integration of genomic datasets with the R/Bioconductor package biomaRt. Nat. Protoc. *4*, 1184–1191. https://doi.org/10.1038/nprot.2009.97.

96. Love, M.I., Huber, W., and Anders, S. (2014). Moderated estimation of fold change and dispersion for RNA-seq data with DESeq2. Genome Biol. *15*, 550. https://doi.org/10.1186/s13059-014-0550-8.

STAR★METHODS

KEY RESOURCES TABLE

REAGENT or RESOURCE	SOURCE	IDENTIFIER
Antibodies		
Guinea Pig anti-MAP2 (1:500)	Alomone Labs	Cat# AGP-046; RRID: AB_2756610
Rabbit anti-EMX1 (1:200)	Sigma	Cat# HPA006421; RRID: AB_1078739
Rat anti-CTIP2 (1:100)	Abcam	Cat# ab18465; RRID: AB_2064130
Rabbit anti-TBR1 (1:200)	Abcam	Cat# ab31940; RRID: AB_2200219
Sheep anti-EOMES (TBR2) (1:200)	Biotechne	Cat# AF6166; RRID: AB_10569705
Mouse anti-DLX2 (1:200)	Santa Cruz	Cat# SC-393879; RRID: AB_3678657
Rabbit anti-NEUROD2 (1:200)	Abcam	Cat# ab104430; RRID: AB_10975628
Chicken anti-GFAP (1:1000)	Abcam	Cat# ab4674; RRID: AB_304558
Goat anti-Nanog (1:100)	R&D systems	Cat# AF1997; RRID: AB_355097
Mouse anti-OCT3/4 (1:200)	Santa Cruz	Cat# SC-5279; RRID: AB_628051
Mouse anti-TRA-1-81 (1:100)	Invitrogen	Cat# MA1-024; RRID: AB_2536706
Mouse anti-VGLUT1 (1:100)	NeuroMab	Cat# 73–066; RRID: AB_10673111
Rabbit anti-GABA (1:500)	Thermo Fisher	Cat# PA5-32241; RRID: AB_2549714
Goat Anti-guinea pig Alexa Fluor 488	Invitrogen	Cat# A11073; RRID: AB_2534117
Goat Anti-rabbit Alexa Fluor 647	Invitrogen	Cat# A21245; RRID: AB_2535813
Goat Anti-rabbit Alexa Fluor 488	Invitrogen	Cat# A11008; RRID: AB_143165
Goat Anti-rat Alexa Fluor 568	Invitrogen	Cat# A11077; RRID: AB_2534121
Goat Anti-rabbit Alexa Fluor 568	Invitrogen	Cat# A11011; RRID: AB_143157
Goat Anti-mouse Alexa Fluor 488	Invitrogen	Cat# A11001; RRID: AB_2534069
Donkey Anti-rabbit Alexa Fluor 488	Invitrogen	Cat# A21206; RRID: AB_2535792
Donkey Anti-sheep Alexa Fluor 568	Invitrogen	Cat# A21099; RRID: AB_2535753
Goat Anti-guinea pig Alexa Fluor 568	Invitrogen	Cat# A11075; RRID: AB_2534119
Donkey Anti-mouse Alexa Fluor 594	Invitrogen	Cat# A21203; RRID: AB_2535789
Donkey Anti-goat Alexa Fluor 488	Thermo Fisher	Cat# A11055; RRID: AB_2534102
Chemicals, peptides, and recombinant proteins		
BrainPhys	StemCell Technologies	05790
NeuroCult SM1	StemCell Technologies	05711
Rolipram	Tocris	0905/10
Forskolin	Tocris	1099/10
Tetrodotoxin Citrate	Abcam	Ab120055
Critical commercial assays		
STEMdiff Cerebral Organoid Kit	Stem Cell Technologies	08570
Neural Tissue Dissociation Kit (P, Papain)	Miltenyi Biotec	130-092-628
Deposited data		
Dissociated organoids RNA-seq	This study	GEO: GSE292624
Raw MEA data and well average data for all parameters	This study	KORDS: 10.18742/c.8097928
Experimental models: Cell lines		
H9 human ESC line (XX)	WiCell	hPSCreg ID: WAe009-A
H1 human ESC line (XY)	WiCell	hPSCreg ID: WAe001-A
KOLF2.1J human iPSC line (XY)	Jackson Laboratory	hPSCreg ID: WTSIi018-B-12
SCTi003-A human iPSC line (XX)	Stem Cell Technologies	hPSCreg ID: SCTi003-A
59XY human iPSC line (XY)	Waldhorn et al.[81]	NA
272XX human iPSC line (XX)	Waldhorn et al.[81]	NA

(Continued on next page)

Continued

REAGENT or RESOURCE	SOURCE	IDENTIFIER
Software and algorithms		
Axis Navigator 3.9.1 with Neural Module	Axion Biosystems	https://www.axionbiosystems.com/products/mea/mea-software/neural-module
Neural Metrics Tool 4.0.5	Axion Biosystems	NA
MEA-NAP 1.10.2	Sit et al.[24]	https://github.com/SAND-Lab/MEA-NAP; https://doi.org/10.7910/DVN/Z14LWA
MATLAB R2023b with toolboxes for running MEA-NAP[24]	MathWorks	https://www.mathworks.com
Other		
Maestro (or equivalent MEA system e.g., Maestro Edge, Maestro Pro)	Axion BioSystems	https://www.axionbiosystems.com/products/mea
24-well 16-electrode MEA plate	Axion BioSystems	M384-tMEA-24w

EXPERIMENTAL MODEL AND STUDY PARTICIPANT DETAILS

Cell line information

Four human induced pluripotent stem cell (hiPSC) lines, SCTi003-A (XX), KOLF2.1J (XY), 272XX (XX), and 59XY (XY) and two human embryonic stem cell (hESC) lines, H9 (XX) and H1 (XY) were used for experiments. To ensure high quality lines with respect to low passage number, characterisation, and standardisation, cells for the project were sourced directly from stem cell banks under material transfer agreements (MTA). The SCTi003-A line was obtained from Stem Cell Technologies (200–0511, Lot# 2206422017) as passage 32. The KOLF2.1J line was obtained from Jackson Laboratory as passage 4. The 59XY and 272XX lines were obtained from the The Hadassah Stem Cell Research Center as passage 25 and passage 20, respectively. The H9 (WA09) and H1 (WA01) lines were obtained from WiCell as passage 29 (Lot# WB67619) and passage 30 (WB34445), respectively. 272XX and 59XY are isogenic control lines with normal chromosomal complements derived from a mosaic Klinefelter's syndrome patient. All other cell lines were derived from control patients with no disease diagnosis. All cell lines had received full consent and ethical approval for research use. Approval for use of hESC lines in this study was obtained from the UK Stem Cell Bank (UKSCB) Steering Committee (reference number SCSC22-03).

All cell lines were expanded and banked, and working stocks were generated from banked vials. Medium from all cell lines was submitted for regular PCR-based mycoplasma testing. Stem cells were stored in liquid nitrogen suspended in freezing medium (10% DMSO in Stemflex medium (Gibco; A3349401)). All stem cells were grown on 6-well plates (NUNC) coated with Geltrex (Geltrex LDEV-Free Reduced Growth Factor Basement Membrane Matrix, Gibco A1413201) diluted 1:100 in DMEM (Sigma; D6421). The stem cells were thawed in a 37°C water bath and the freezing medium diluted with DMEM. The cells were then pelleted at 900 RPM for two minutes and resuspended in Stemflex medium with 1:100 Revitacell (Gibco; A2644501). The cells were kept in hypoxic conditions at 37°C and 5% CO_2, 5% O_2. After 24 h, the medium was replaced with Stemflex medium, and full media exchanges were performed every 48 h. The stem cells were passaged once 70–80% confluent by washing with HBSS and then incubating for 3 min in room temperature Versene (Gibco; 15040066). The Versene was aspirated and replaced with Stemflex medium, the cells were dislodged by pipetting medium over the cells. The cells were then added to the appropriate volume of medium for splitting into new Geltrex-coated plates (between 1:2 to 1:6). The stem cells were monitored daily using brightfield microscopy (EVOS XL). Wells with 70–80% confluence and no visible signs of spontaneous differentiation were used to generate cerebral organoids. iPSCs used to generate organoids were not allowed to exceed passage 35, and ESCs were not allowed to exceed passage 48.

In addition to cell line quality checks conducted as part of cell line sourcing additional checks for pluripotency and common karyotypic abnormalities were done following established guidelines.[81] A subset of undifferentiated stem cells from the working stock was grown on glass coverslips and immunocytochemistry was conducted to check expression of the pluripotency markers NANOG, POU5F1, and PODXL. DNA was extracted from organoids from the highest passage batches of all the cell lines using a Genomic DNA Extraction Kit (Monarch; T3010). The DNA samples were tested for the most common genetic abnormalities that can arise during stem cell passaging and expansion using the hPSC Genetic Analysis Kit (Stem Cell Technologies; 07550) according to the manufacturer's protocol.

METHOD DETAILS

Generation of unguided cerebral organoids

Cerebral organoids were generated using the commercially available STEMdiff Cerebral Organoid Kit (Stem Cell Technologies; 08570) and associated protocol (Stem Cell Technologies Document #DX21849). To reduce batch variation, organoids were generated from cell line stocks of similar passage number, and multiple cell lines were differentiated in parallel by the same researcher. Organoids generated on the same day from the same passage number of stem cells were considered part of the same batch.

The organoid kit protocol was modified to allow for long-term culture of cerebral organoids using a published protocol.[82] The following changes to the manufacturer's protocol were made: the embryoid body (EB) seeding medium was supplemented with a higher concentration of ROCK inhibitor (50 μM) (as recommended in Giandomenico et al.[82]). Smaller EBs were generated using fewer cells (~1000 cells per EB) as we found that this worked well for the cell lines used. Geltrex was used instead of Matrigel in all steps of the protocol. In brief, the protocol was as follows: First, embryoid bodies (EBs) were generated by seeding stem cells into a 96-well round-bottom ultra-low attachment plate (Corning; 7007) at 0 days *in vitro* (DIV). The EBs were kept in hypoxic conditions at 37°C and 5% CO_2, 5% O_2 until 5 DIV. At 5 DIV, the EBs were transferred to induction medium in a 24-well ultra-low attachment plate (Corning; 3473) and hereafter incubated in normoxic conditions at 37°C and 5% CO_2. At 7 DIV, EBs were embedded in Geltrex and transferred to expansion medium until 10 DIV. From 10 DIV onwards, organoids were maintained in maturation medium consisting of STEMdiff Cerebral Organoid Basal Medium 2 and STEMdiff Cerebral Organoid Supplement E (Stem Cell Technologies; 08571). The organoids were placed on an orbital shaker (Infors; Celltron) in the incubator at 37°C and 5% CO_2, with the shaker set to 57 revolutions per minute. The Geltrex was removed from the encapsulated organoids at ~12 DIV using a 10 mL stripette. Organoids were grown in 50 mm dishes and fed by a full medium change of 5 mL maturation medium twice a week. The organoids were split (e.g., organoids were divided between two dishes) when the medium turned yellow between feedings. The maturation medium was supplemented with 2:100 Geltrex during feeding from 30 DIV until 50 DIV to promote growth of the cortical plate. Antibiotics were not used in any stage of the protocol. The organoids were grown until 45–47 DIV when they were dissociated. Quality control checks were done throughout the organoid generation and maturation protocol by regular monitoring of the development of the organoids using brightfield microscopy. Quality criteria were based on the recommendations provided by Stem Cell Technologies and additionally in the published protocol[44] – these were as follows: Stem cells without any obvious signs of differentiation were used and if EBs failed to form by day 3 of the protocol, the experiment was terminated, and a new vial of stem cells was used. Additional quality control steps included checking that the EB borders showed clearing prior to induction, checking for the formation of radially organized neuroepithelium following induction, checking for formation of neurepithelial buds during expansion, and removal of any organoids showing excessive cyst formation, migratory cells, or disintegration.

Dissociation of cerebral organoids for culture on MEA

Whole organoids were dissociated at 45–47 DIV using the Neural Tissue Dissociation Kit (P, Papain) (Miltenyi Biotec; 130-092-628). To reduce the impact of inter-organoid variability, we pooled multiple organoids per batch together (a minimum of 6 organoids). For each batch and cell line, six organoids from the same batch were transferred to a Petri dish (Thermo; 150462) containing 5 mL HBSS with calcium and magnesium (+/+, Gibco 14025–092) using a cut 1 mL pipette. The organoids were then cut into several pieces using a sterile scalpel (Swann-Morton 0501), visually targeting the cortical lobe regions of the organoids. The cut organoids were gently washed in the Petri dish using circular movements three times and were then transferred to a 15 mL falcon tube and the supernatant was aspirated. A volume of 2 mL of preheated enzyme mix 1 (50 μL Enzyme P into 1900 μL Buffer X) was added to the organoids to initiate papain-based digestion. The organoids were incubated at 37°C for 15 min. Enzyme Mix 2 (DNAse mix containing 20 μl of Buffer Y and 10 μl of Enzyme A) was then added to the reaction. The organoids were triturated approximately 20 times using a cut 1000 μL pipette tip and then a standard 1000 μL tip was used to break up the organoid pieces. This process was repeated every 5 min of incubation up to a total of ~3 times until the solution was cloudy and no tissue pieces or aggregates were observable. Any remaining clumps were removed by filtering through a 40 μm nylon mesh sterile strainer (Fisher brand; 22363547). The strainer was pre-wetted with HBSS without calcium and magnesium (−/−, 1 mL, Gibco; 14170–070) prior to the addition of the organoid cell suspension. The filter was then washed with 2 mL of HBSS (−/−). The cells were pelleted in a centrifuge, the supernatant aspirated, and the cells resuspended in 5 mL of HBSS (−/−) and pelleted again. The cells were then resuspended in FBS recovery medium with 100 μg/mL laminin to achieve a concentration of 7.5 million cells/mL. FBS recovery medium consisted of 445 mL high-glucose DMEM supplemented with GlutaMAX (Gibco; 10569010), 50 mL FBS (Gibco; 10270–106) and 5 mL of 50% (wt/vol) glucose solution[82] without antibiotic-antimycotic.

Dissociated organoid plating and MEA culture

MEA multi-well plates with 16 embedded microelectrodes per well (Axion; M384-tMEA-24w) for culturing cells from dissociated organoids were coated with 0.1% Poly(ethyleneimine) solution (PEI) for 60 min at 37°C, 5% CO_2. The PEI was deposited on the area of the electrodes as 10 μL droplets. The PEI was rinsed with 200 μL of sterile DI water (ddH2O) 4 times, and the plate was allowed to air-dry overnight. The MEA plate was then coated with 9.6 μg/mL laminin (Sigma; L2020-1MG) in DMEM (Sigma; D6421) for 4 h at 37°C, 5% CO_2 and then rinsed three times with HBSS (−/−). A volume of 10 μL of cell suspension (approximately 75,000 cells per well) was applied to the recording electrodes on the electrode array and the plated cells were incubated 60 min at 37°C, 5% CO_2. FBS recovery medium was then slowly applied to the wells to a final volume of 0.5 mL. Half media changes were performed every other day with BrainPhys+SM1 (BrainPhys (StemCell Technologies; 05790) supplemented with 200 μL/9.8 mL NeuroCult SM1 (StemCell Technologies; 05711)). The BrainPhys+SM1 medium was also supplemented with 2 μg/mL laminin weekly.

MEA recording

Continuous MEA recordings were made across all electrodes in real time using the Axion Maestro and Axis Navigator software (12.5 kHz sampling rate, 200–3000 Hz band-pass) and were stored as.raw files. The temperature and CO_2 were allowed to stabilise at 37°C

and 5% prior to recording after inserting the plate into the Maestro. Recordings were always made prior to any media changes, or after a minimum of two hours after a medium change if required by the treatment protocol. For acute treatment experiments, a 5-min baseline recording was taken prior to adding the treatment. Recordings were made over 5-min and subsequently analyzed. For comparison of spontaneous activity between cell lines and batches of dissociated organoids from each cell line, daily five-minute recordings were taken from 6 DPD to 37 DPD in one batch of H1 dissociated organoids, one batch of H9 dissociated organoids, two batches of SCTi003-A dissociated organoids, and 3 batches of KOLF2.1J dissociated organoids. Recordings consisted of N = 12 wells per batch and cell line, except for the third batch of KOLF2.1J organoids, where 48 wells were used, split across two plates. Half of the wells were exposed to 17β-Estradiol from 29 DPD onwards, control recordings from 30 DPD onwards thus consisted of N = 6 wells per batch and cell line.

Treatment experiments

Tetrodotoxin treatment

Tetrodotoxin Citrate stock was made up to 10mM in H_2O and then diluted in BrainPhys media. After spiking into treated wells to make up to a final concentration of 1 μM, the plate was transferred to the Maestro and recordings were made 5 min after equilibration in the machine.

17β-estradiol treatment

17β-Estradiol powder (E2758-250MG; Sigma) was dissolved in 1 mL absolute ethanol and a 20 μg/mL stock solution made by adding DMEM/F-12 phenol red-free (PR-F) (Gibco; 21041025). Vehicle stock solution was made in the same manner, but without 17β-Estradiol powder. The 17β-Estradiol and vehicle stocks were stored as single-use aliquots at −20°C for up to 1 month. Diluted treatment solutions were prepared freshly each day, by sequential dilution of the stock, first to a 10 μM solution in DMEM PR-F, then to a working stock of 1 μM in BrainPhys+SM1 PR-F. 1 μM 17β-Estradiol or vehicle solution was added to PR-F cell culture medium, with a final concentration of 10 nM. Dissociated organoids were transitioned to medium without phenol red at least 2 days prior to commencing treatment experiments. 17β-estradiol or vehicle were spiked directly into the dissociated cultures every day following recording (6 μL spike to 600 μL of medium in each 24-well plate).

chLTP

chLTP treatment was conducted according to the method described by Pré et al.[49] for iPSC neurons. The chLTP experiment was done using KOLF2.1J dissociated organoids from one batch of nine pooled organoids in two replicates on two different days, with 6 wells per condition per replicate (12 total wells for chLTP and 12 vehicle wells). The chLTP treatment consisted of Rolipram (Tocris; 0905/10), stock prepared as 25 mM in DMSO, and Forskolin (Tocris; 1099/10), stock prepared as 50 mM in DMSO. On the day of treatment, a full medium change with 600 μL media per well was done two hours prior to the start of the treatment. Next, a baseline recording was taken and 17β-estradiol to a 10 nM concentration or vehicle (containing the same concentration of ethanol) were added for 15 min. After 15 min of pre-treatment, 30 μL medium was removed from each well of the MEA and 30 μL of 20x chLTP treatment solution was added, consisting of 1000 μM Forskolin and 2 μM Rolipram diluted in Brainphys media. The vehicle condition treatment consisted of the same amount of DMSO in Brainphys, but without Forskolin and Rolipram. 30 min after chLTP treatment, electrical activity was recorded for 5 min and a washout was done to remove the treatment: all medium was removed, followed by addition of 200 μL BrainPhys+SM1, all medium was removed again, finally 600 μL BrainPhys+SM1 media were added.

Immunocytochemistry

Dissociated organoids were grown on round No. 1.5 coverslips placed in 12-well plates plates or in 96-well PhenoPlates (Revvity). The cells were cultured as normal and the cells were then fixed in 4% PFA with 4% sucrose in PBS with calcium and magnesium (+/+, Gibco 14040117) for 10 min at room temperature, washed twice in PBS (+/+). Cells on glass coverslips were then additionally fixed in ice-cold methanol at 4°C for 10 min. The cells were then washed 2x in PBS (+/+) and stored in PBS (+/+) at 4°C until used. For staining, cells were permeabilised and blocked in 2% NGS or 2% NDS in PBS (+/+) with 0.1% Triton X- for 1.5 h. Primary antibodies were diluted in the same buffer, but without Triton X-. Cells were incubated with primary antibodies at 4°C overnight then washed 3x with PBS (+/+) for 15 min. Plates or coverslips were then incubated with secondary antibodies diluted 1:1000 in 2% NGS/NDS for 1 h at room temperature in the dark. The coverslips were then washed 3x with PBS (+/+) for 10 min and mounted onto microscope slides with Prolong Gold with DAPI as mounting media. The slides were allowed to cure overnight and were then sealed with nail polish. For 96-well plates, DAPI was added in the last 5 min of the secondary antibody incubation and 3x washes with PBS (+/+) were performed before imaging.

Microscopy

Confocal microscopy was used to image organoid sections stained using IHC for regional and cell type identity markers and cells from dissociated organoids stained using immunocytochemistry. Confocal images were acquired using an upright Nikon A1R confocal microscope with a 20× air objective, (N.A. 0.75) (www.kclwcic.co.uk/multiphoton). To image an entire section, a focus surface was set using the DAPI channel and the "scan large image" function was used to generate an assembled tile scan of one Z plane. Cells on coverslips were imaged as multiple regions of interest with a z stack encompassing the cell layer. All acquisition parameters (including z stack thickness, if applicable) were kept constant within an experiment.

High-content imaging was used to image 96-well plates containing dissociated organoids. Images were acquired using Opera Phenix High-content System with a 20× air objective. 8 fields of view were acquired in each well with 3 z stack of 1.5 μm distance.

RNA-seq

Cell lysis and RNA extraction

To extract total RNA from cells grown on the MEA from dissociated organoids, the medium was aspirated and 250 μL/well of accutase were added and the plate was incubated for 5 min at 37°C, 5% CO2. The cells with accutase from vehicle treatment condition were pipetted and pooled into a tube and an equal volume of HBSS was added. The cells were centrifuged at 1250 RPM and the medium was aspirated. The cells were resuspended in 1 mL TRIzol, pipetted vigorously and transferred to a RNAse-free centrifuge tube. Each TRIzol sample containing lysed cells was incubated at room temperature for five minutes. TRIzol samples were stored at −80°C until RNA could be extracted. To extract total RNA, samples were thawed at room temperature and incubated for 5 min at room temperature once thawed. 200 μL of chloroform was added to each sample, and the tubes were inverted and incubated for three minutes at room temperature. Finally, the sample was centrifuged at 10000 RPM for 5 min at 4°C and the aqueous phase was transferred to a new tube. 1 μL of glycogen and 500 μL isopropanol were added to the aqueous phase to increase RNA purity and yield. The sample was then inverted to mix, incubated for 15 min at room temperature, and centrifuged at 13000 RPM for 15 min at 4°C. The supernatant was discarded, and the pellet was washed by addition of 1 mL of 80% ethanol, centrifugation at 13000 RPM for 5 min at 4°C, and removal of the ethanol. Each pellet was air dried until transparent and resuspended in 30 μL RNAse-free H_2O. To clean the RNA, the samples were then precipitated with 3M sodium acetate (Sigma S2889). 3 μL of sodium acetate and 90 μL 100% molecular-grade ethanol were added and the sample was incubated at −80°C overnight. Finally, the sample was centrifuged at 13000 RPM for 15 min at 4°C and the supernatant was discarded. The pellet was washed with 80% ethanol two times, air dried until transparent, and then resuspended in 30 μL RNAse-free H_2O. The RNA and protein concentration in each sample was measured using absorbance with a NanoDrop OneC spectrophotometer (Thermo Scientific ND-ONEC-W). The RNA was stored at −80°C.

Bulk RNA sequencing

Purified RNA samples were sent for sequencing to Genewiz Inc (Azenta Life Sciences), who conducted additional quality control to check RNA integrity, size, and concentration using an RNA Qubit and Fragment Analyzer. Illumina sequencing libraries with PolyA selection were prepared in-house by Azenta. Sequencing was carried out using the Illumina NovaSeq in a 2 × 150bp configuration with an estimated data output of ~20M paired-end reads per sample. Raw data was obtained from Azenta as FASTQ files.

QUANTIFICATION AND STATISTICAL ANALYSIS

MEA data analysis

For data analysis, the.spk file was exported using Axis Navigator to the Neural Metrics Tool, where an analysis configuration was applied to extract individual MEA parameters (Table S1). The definitions of the MEA parameters (Table S1) are based on the definitions in the Axis Navigator User Guide, version 3.5.[83] The same analysis configuration was used across all recordings. To accommodate the lower network activity observed in organoids, we adjusted the default analysis configuration settings for bursting and network bursting. The analysis configuration used was as follows.

Configuration	Setting
Adaptive Threshold (SDs)	5.5
Active Electrode Criterion (spks/min)	5
Remove Coincident Artifacts	FALSE
Electrode Burst Settings	
Algorithm	Poisson Surprise
Min Surprise	3
Network Burst Settings	
Algorithm	ISI Threshold
Min # of Spikes	35
Max inter-spike interval (ISI) (ms)	100
Min # of Electrodes (%)	25
Synchrony Settings	
Synchrony Window	20
Average Network Burst Analysis Settings	
Network Burst Window Start (ms)	0
Network Burst Window End (ms)	500
Bin Size (ms)	1

"Recommended metrics", which include all standard parameters were exported to CSV files for statistical analysis in R (version 4.2.2). Raster plots of MEA activity with spike histograms were generated using Axis Navigator (Axion, version 3.9.1) and Neural Metrics Tool (Axion, version 4.0.5). Raw spike plots were generated using Axis Navigator. We used 6 and 37 DPD, along with three intermediate timepoints (15, 22, and 29 DPD), for more in-depth analysis of temporal changes in network activity. For the purposes of a consistent comparison of spontaneous activity, we focused on four key parameters extracted from the Neural Metrics Tool across experiments, these being: *mean firing rate*, *number of bursts*, *area under normalised cross-correlation*, and *number of network bursts* (Table S1). These parameters each represent the primary parameter from each level of spontaneous activity (activity metrics, electrode burst metrics, network burst metrics, and synchrony metrics). Mean firing rate (the number of spikes over time (Hz)), was used as a general measure of spontaneous activity. Number of bursts was measured as the total number of single-electrode bursts across all electrodes in a well. Bursts were detected using a Poisson Surprise algorithm in the Axion Neural Metrics Tool, where neurons are assumed to fire according to a Poisson distribution, and the probability of a cluster of spikes appearing by chance is assessed using a "surprise" threshold.[83,84] Area under normalised cross-correlation was used as a measure of synchrony across multiple electrodes. Area under normalised cross-correlation is the area under the curve of a pooled cross-correlogram across all unique combinations of electrodes in a well, normalised to auto cross-correlations (cross-correlation of an electrode to itself) to reduce effects of variability in firing at individual electrodes.[61,83] Cross correlation measures the probability of a spike in one channel relative to a reference channel in a pair, with each spike being treated as time = 0 in the reference, and surrounding spikes in the paired electrode being binned in a histogram. This parameter represents the likelihood of spikes happening together in pairs of electrodes and has been used as a readout of synchrony in previous organoid studies.[69] As another measure of network activity, we measured the number of network bursts across all wells using an inter-spike interval (ISI) threshold method[22,83] with network bursts logged upon detecting at least 35 spikes across all electrodes in the well, each separated by an ISI of no more than 100 milliseconds with at least 25 percent of electrodes participating in the network burst. This parameter considers both the mean firing rate and the firing patterns across electrodes, providing an overview of the overall activity and synchrony of a neuronal network, and thus how coordinated or irregular the firing is across the network.

For functional network analysis, MEA network plots, and raster plots were plotted using MATLAB (version R2023b) and the MATLAB MEA network analysis pipeline (MEA-NAP, version 1.10.2).[24] Waveform plots were generated using Axis Navigator. Analysis in MEA-NAP was done directly on.raw files exported from Axis Navigator (Axion). Raw files were first converted to.mat files using the file conversion tab in MEA-NAP. MEA-NAP was run using recommended settings, with sampling frequency set to 12500 Hz, potential difference units set to V, channel layout set to Axion 16, and wavelet cost of -0.12.[24] Spikes were detected using a template-based method, where a wavelet transform was used to identify spikes similar to a template wavelet (bior1.5 template). A spike time tiling lag (STTC) of 50 ms was used to determine correlation between spikes at different electrodes.[85] A 50 ms STTC lag was used to take into account the larger spacing of electrodes, in order to detect network activity where not all neurons firing are detected. To remove correlations that occur by chance, the MEA-NAP pipeline detects significant functional connections using probabilistic thresholding. Network metrics exported by MEA-NAP were used for statistical analysis in R. We focused on analysis of two basic node-level parameters extracted from MEA-NAP across experiments, these being mean node degree and mean edge weight (Table S1).

Unless otherwise stated, all MEA plots were generated using *ggplot2*[86] in R from well average data exported from the Neural Metrics Tool or recording-level data for network activity exported from MEA-NAP.

Statistical analysis

Given that each well generated a unique neuronal network, each well was considered a biological replicate for the purpose of statistics, with batch and cell line, where applicable, used as covariates. Statistical analysis was performed using R version 4.2.2 in R Studio. All measured wells were included in the statistical analysis. Statistical analysis results are presented in Table S3, with significant effects being ones where $p < 0.05$. All analysis for single-electrode parameters was performed on the well averages for all wells, e.g., the average value for all electrodes in a well.

For analysis of activity over time, the effect of timepoint was analyzed using a mixed-effects model with cell line as a fixed effect, followed by estimated-marginal means post-hoc comparisons for each cell line and DPD combination with Bonferroni correction for multiple comparisons (Table S3). This approach was used to account for the repeated measures design and fewer control wells at the 37 DPD timepoint.

For analysis of cell line differences at 37 DPD, analysis of variance (ANOVA) was done for each of six core parameters for the model *parameter* ~ *line type (ESC or iPSC)* + *Cell line* or *parameter* ~ *sex (XX or XY)* + *Cell line*. ANOVA tables are presented in Table S3, with significant effects being ones where $p < 0.05$. Post hoc tests for pairwise comparisons between cell lines were performed using Tukey's Honest Significant Difference method (Table S3).

For analysis of effects of chLTP or E2 (Table S3), we calculated the difference in the well average value of each parameter between baseline and 6 h following chLTP, Vehicle, E2, or chLTP+E2 treatment, with $N = 12$ per condition across two replicates from the same batch. Analysis of variance was done for each of six core parameters for the model *difference from baseline* ~ *Condition* + *Replicate*. We selected this model over a model with interaction of replicate and condition using the Akaike Information Criterion (AIC). For parameters with a significant effect of condition, post hoc tests were performed using Tukey's Honest Significant Difference method (Table S3). The same analysis method was used for effects of E2 compared to vehicle, where we compared the difference from

baseline at 24 h following the first treatment and 8 days following the first treatment (7 days of daily treatment) in the vehicle condition compared to E2. The 7-day E2/vehicle treatment experiment was performed in dissociated organoids from three cell lines (H1, H9, KOLF2.1J, and SCTi003-A), where $N = 6$ per condition per line and batch. Two batches were used for the KOLF2.1J and SCTi003-A lines.

For all linear regression analysis, diagnostic plots of residuals were plotted for each model to check assumptions of equal variance, outliers, normal distribution, and linearity. Data for some parameters in the temporal analysis, as noted in Table S3, was log-transformed as log(1+parameter) prior to statistical analysis.

Dimensionality reduction analysis

For dimensionality reduction analysis, we excluded wells without active electrodes (less than 5 spikes/minute). The following parameters were used for dimensionality reduction and clustering: network size, AUNCC, mean percentage of spikes in bursts, network density, global efficiency, inter-spike interval coefficient of variation, mean firing rate, mean node degree, number of network bursts, mean node strength, modularity score, mean significant edge weight. We selected parameters that could be measured across all wells and timepoints in active wells. Thus, we did not include parameters such as network burst duration, as such parameters are only measurable in wells with network bursting present. We also excluded parameters that were highly correlated. UMAP plots were generated using the umap R package.[87] Data for PCA analysis was normalized using the 'scale()' R function and principal components were calculated using the 'princomp()' function. Plots of variables were plotted using the factorextra[88] package. Hierarchical clustering of the normalized parameters was performed using the 'dist()' R function to generate a matrix of euclidean distances, followed by clustering using 'hclust()' (average linkage method). PCA plots, dendrograms, and UMAPs were plotted using ggplot2.

Analysis of dissociated organoid culture morphology

To monitor morphology over time, a minimum of four wells per line and batch of dissociated organoids were imaged at 7, 13, 27, and 37 days after dissociation using an EVOS XL brightfield confocal microscope at 10× magnification. To quantify the ratio of sparse areas in the well with a cell monolayer to dense areas with cell clusters, a multi-class segmentation was applied to the brightfield images taken at 37 days post dissociation using the pixel classification function in Ilastik[89](1.4.0.post1), by training an Ilastik classification model on a subset of the images (11 training images). Areas of the well were segmented into dense areas, sparse areas, and electrodes. Segmented tiff format images were imported into FIJI 2.14.0/1.54F and the *glasbey* look-up table (LUT) was applied. Each image was converted to RGB, the channels split, inverted, converted to a mask, and the mask was used to create a selection, which was measured. A "cluster ratio" was calculated as the sparse area divided by the dense area. This ratio method uses the same principle as the method described by Hörberg et al.,[70] but uses a different segmentation strategy. Segmented images for figures had the *glasbey inverted* LUT applied to be colourblind friendly.

Bulk RNA-seq analysis

The FASTQC tool (version 0.12.1) was used to do initial read quality control on the FASTQ files.[90] FASTQ files were processed using command line tools on the King's College London CREATE high-performance computing cluster. Alignment of reads to the human genome (Assembly GRCh38.p14, accession GCF_000001405.40, NCBI RefSeq) was performed using STAR (version 2.7.6a).[91] After alignment, the output BAM files were sorted by chromosome and position coordinates using Samtools (version 1.17) and Picard (version 2.26.2) was used to mark duplicates in the BAM files.[92,93] The duplicate checked BAMs were downloaded from CREATE and subsequent analysis was carried out in R (version 4.2.2). A raw counts table was generated using the *featureCounts* function from the Rsubread package.[94] Additional attributes were annotated using the *getBM* function from the *biomaRt* package, including Entrezgene ID, HGNC symbols, ENSEMBL gene IDs, gene size, chromosome and position.[95] Differential gene expression analysis was performed using the *DESeq2* package.[96] Heatmaps were generated using the *pheatmap* R package (version 1.0.12), dendrogram nodes were sorted based on subtree distance using the *dendsort* R package (version 0.3.4).

Analysis of estimated organoid regional identity

The *VoxHunt*[63] package was used estimate the proportions of regional identities in each dissociated organoid sample. Gene lists representing brain regions were extracted from Allen Brain Atlas E13 *in situ* hybridization mouse data and were used to deconvolve the RNA-seq data using the *VoxHunt deconvolute* function. RNA-seq data was inputted as DESeq2 normalised counts for each sample.

Cell Reports Methods, Volume *6*

Supplemental information

Electrophysiological development and functional plasticity in dissociated human cerebral organoids across multiple cell lines

Adam Pavlinek, Sara Guerrisi, Kara O'Driscoll, Lucia Dutan Polit, Roland Nagy, APEX consortium, Madeline A. Lancaster, Anthony C. Vernon, and Deepak P. Srivastava

Table S1. MEA parameter definitions. Related to STAR methods.

Parameter	Definition
**Spiking parameters**	
Mean Firing Rate (MFR)[a, b]	(Number of Spikes)/(Analysis duration) in Hz
Inter-spike interval (ISI)	The time between spikes.
ISI Coefficient of Variation[b]	(Standard deviation (SD) of ISI)/(mean ISI). Measure of spike regularity.
Network ISI Coefficient of Variation	Coefficient of variation (standard deviation/mean) of the ISI for all spikes on all electrodes in a well. Measure of spike regularity across the network, captures the distribution of spiking such that 0 indicates spikes perfectly distributed and >1 indicates network bursting.
Number of Active Electrodes	Number of electrodes with activity greater than the minimum spike rate of 5spks/min.
Weighted MFR	Mean firing rate using data from active electrodes only.
**Single electrode bursting parameters**	
Number of Bursts[a]	Total number of single-electrode bursts over a given duration.
Mean ISI within Burst	Average ISI, for spikes in a single electrode burst. Measure of burst intensity; smaller values mean more intense bursts.
Median ISI within Burst	Median ISI for spikes in a single electrode burst.
Inter-burst Interval (IBI)	Average time between the start of single-electrode bursts.
IBI Coefficient of Variation	The coefficient of variation (standard deviation/mean) of the inter-burst interval, the time between single-electrode bursts. This is a measure of single electrode burst regularity.
Burst Percentage[b]	(Number of spikes in single-electrode bursts)/(total number of spikes)*100
**Network bursting parameters**	
Network Burst Number (NBN)[a,b]	Total number of network bursts over a given duration.
Network Burst Duration	Average time from the first spike to last spike in a network burst.
Number of Spikes per Network Burst	Average number of spikes in a network burst.
Number of Electrodes Participating in a Burst	Average number of electrodes with activity during a network burst.
Number of Spikes per Network Burst per Channel	(Average number of spikes per burst)/(number of electrodes participating in that burst)
Network Burst Percentage	(Number of spikes in network bursts)/(total number of spikes)*100
Network IBI Coefficient of Variation	The coefficient of variation (standard deviation/average) for the inter-network burst interval. A measure of network burst regularity.

Network Normalized Duration IQR	Interquartile range of network burst durations. Provides a measure of network burst duration regularity. If the middle 50% of network bursts are approximately the same duration, this value will be small, whereas, if the network bursts vary widely in duration, this range will be large.
**Synchrony parameters**	
Area Under Normalized Cross-Correlation (AUNCC)[a, b]	Area under the well-wide pooled inter-electrode cross-correlation normalized to the autocorrelations. Higher areas indicate greater synchrony.
**Functional connectivity parameters**	
Mean Node Degree (MND)[a, b, c]	Mean number of significant connections (edges) of each node with other nodes (electrodes) in the network, determined using probabilistic thresholding.
Mean Edge Weight[a, b, c]	Mean Significant Edge Weight. Edge weight is the strength of connectivity between two nodes, calculated using a spike-time tiling coefficient of 50ms.
Modularity Score (Q)[b, c]	A value between -0.5 and 1 that describes how well a network has been partitioned.
Mean Node Strength (NSmean)[b, c]	Mean of node strength for all nodes in a well, where node strength is the sum of the edge weights for each node.
Global Efficiency (Eglob)[b, c]	Efficiency of parallel information transfer between nodes in the network.
Controllability[b, c]	Mean Average Controllability, metric of how much influence a node has over the overall network activity.
Network Density[b, c]	Number of connections (edges) as a proportion (%) of the total possible connections that can be formed in the network.
Network size (aN)[b, c]	Number of active electrodes in MEA-NAP pipeline

Parameter definitions for parameters discussed in the study. Definitions for parameters calculated using the Axion Neural Metrics Tool are taken from the Axis Navigator User Guide, Axion BioSystems [1]. Definitions for parameters calculated using MEA-NAP are taken from Table 1 of Sit et al. [2].

[a]Core parameter used for analysis of activity and network characteristics
[b]Parameter included in main dimensionality reduction analyses
[c]Parameter calculated using MEA-NAP, Sit *et al.* [2]

Table S2. Comparison of network parameters between two KOLF2.1J batches. Related to Figure 4.

Parameter	Batch 1 (mean ± SD)	Batch 2 (mean ± SD)
Network Burst Number	10.33 ± 3.08	2.83 ± 1.11
Number of Spikes per Network Burst - Avg	108.56 ± 43.19	686.42 ± 205.59
Number of Elecs Participating in Burst - Avg	7.69 ± 1.61	12.63 ± 2.09
Number of Spikes per Network Burst per Channel - Avg	13.99 ± 5.7	53.4 ± 13.3
Mean ISI within Network Burst - Avg (sec)	0.0158 ± 0.0041	0.0030 ± 0.0024
Burst Percentage - Avg	45.23 ± 8.46	72.29 ± 11.58
Network Burst Duration - Avg (sec)	1.29 ± 0.2	1.49 ± 0.29
Median/Mean ISI within Network Burst - Avg	0.66 ± 0.04	0.37 ± 0.07

Values are the mean of all well averages ± standard deviation at 30 DPD.

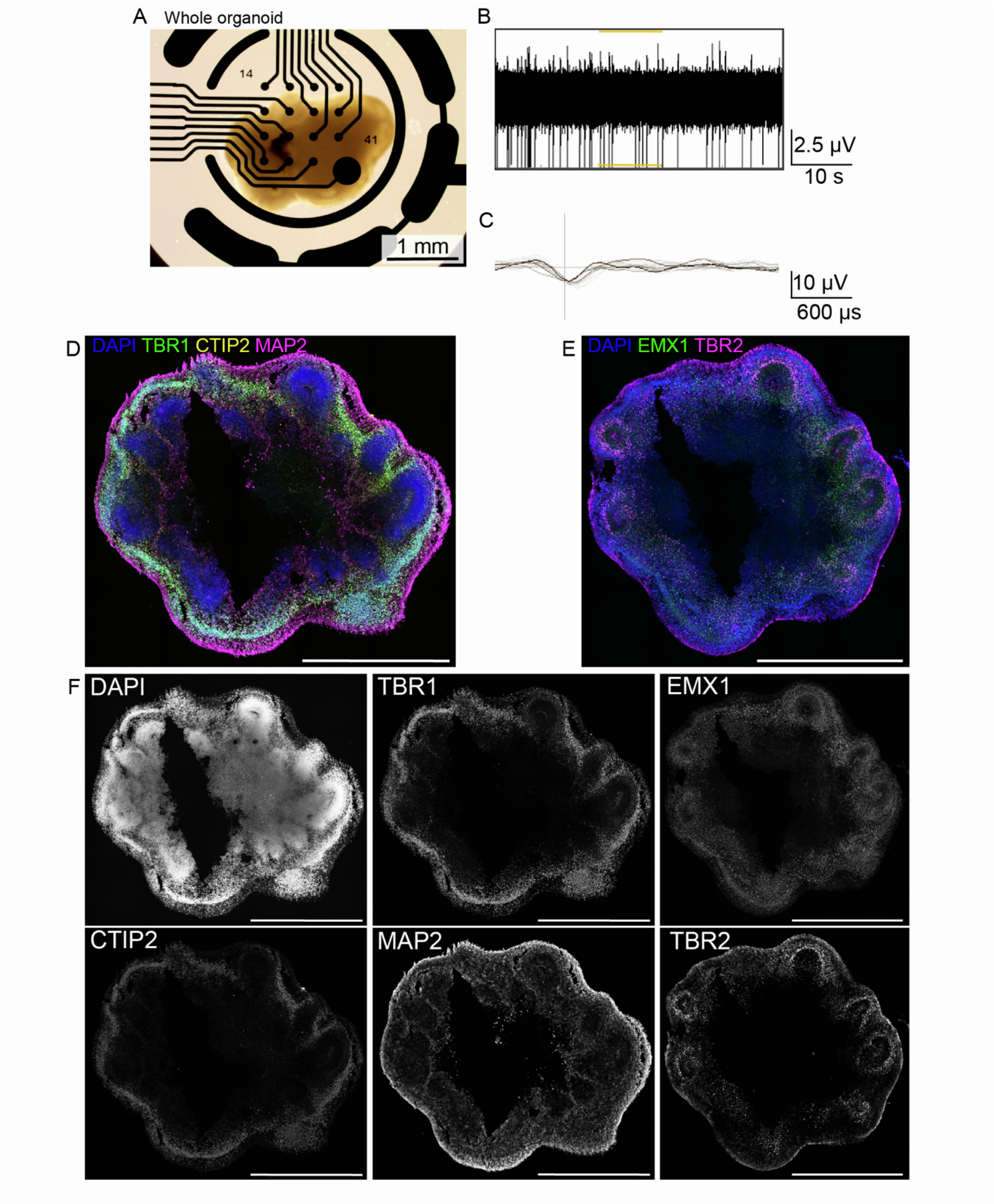

Figure S1. Immunohistochemistry and MEA recording of cerebral organoids at 40 DIV. Related to Figure 1.
(A) Brightfield image of whole cerebral organoid attached to a laminin-coated MEA plate.

(B-C) Snapshot of recording from H9 line whole organoid at 40 DIV showing unsorted overlaid spike waveforms recorded at a single electrode. Waveforms (C) correspond to individual spikes above the 5.5

standard deviation (SD) threshold (B). The darkest waveform represents the most recent spike recorded at the electrode. Waveforms were generated using Axis Navigator.

(D-F) Confocal tile scans of two adjacent serial sections of a representative SCTi003-A line organoid at 40 DIV. Immunostaining for dorsal forebrain markers EMX1 and TBR2 (E), MAP2 and dorsal forebrain markers CTIP2 and TBR1 (D). Corresponding grayscale images for each channel (F). Brightness and contrast were adjusted to allow markers to be distinguished. All scale bars represent 1 mm.

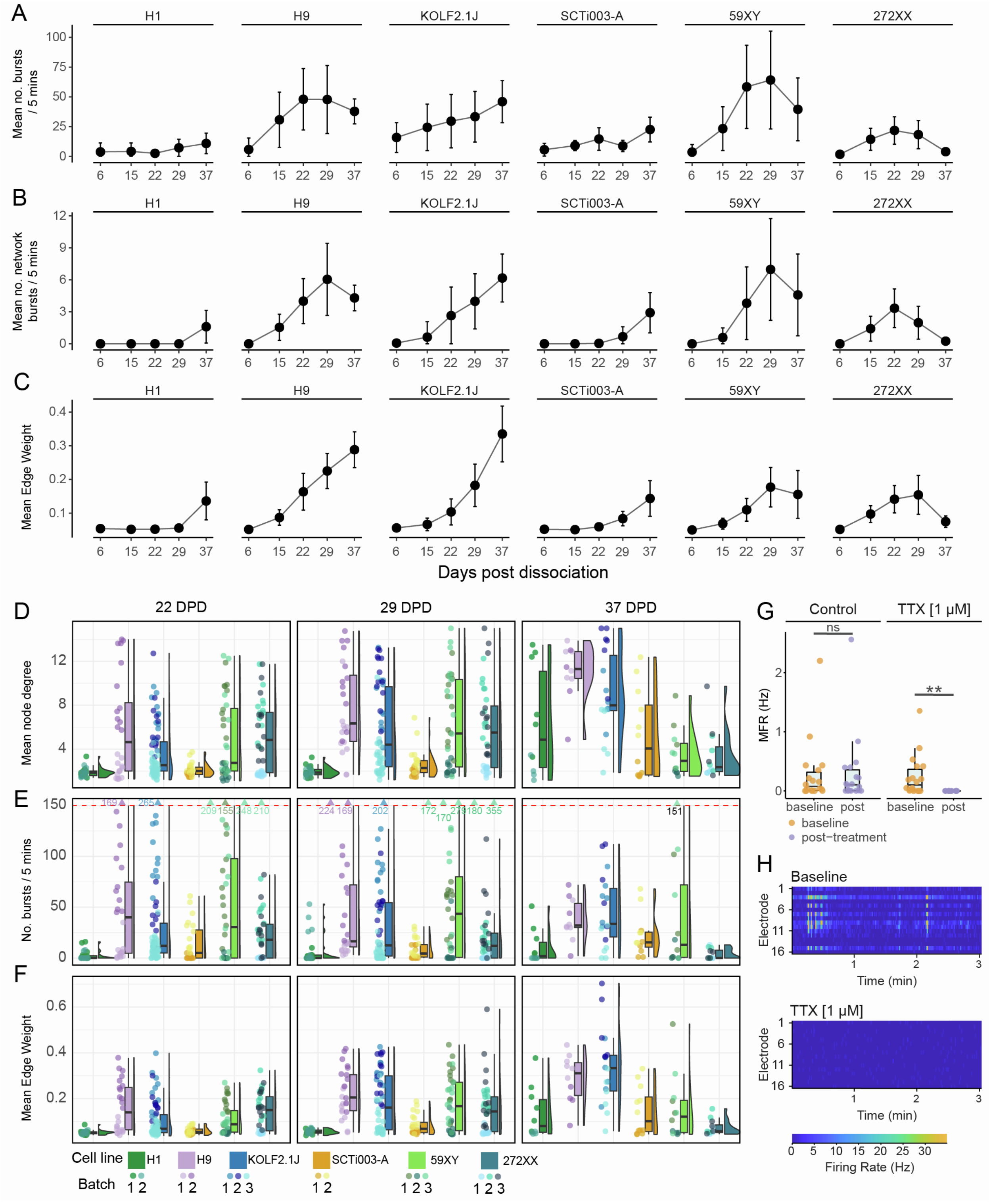

Figure S2. Bursting and network connectivity increases over time, related to Figure 2.
(A-C) Line graphs of mean number of bursts (A), mean network burst number (B), and mean edge weight (C) at 6-, 15-, 22-, 29-, and 37-days post dissociation (DPD). Points represent the mean of well averages ± SD. Full statistical analyses for these parameters are in table S3.

(D-F) Box and violin plots of the distribution, min, max, median, and interquartile range of mean node degree (D), mean number of bursts (E), and mean edge weight (F) per well at 22, 29, and 37 DPD. Each point represents the mean of one well across 5 minutes of recording. The colors of each point indicate the batch and cell line. The boxplot color indicates the cell line, ordered from left to right as H1, H9, KOLF2.1J, SCTi003-A, 59XY, and 272XX. For clarity, number of bursts (E) is capped at 150, points with >150 bursts are shown above the dotted line and annotated with their actual values.

(G) Boxplots of MFR at baseline and immediately after addition of 1µM TTX or control in the same plate across three plates (N=6 wells per condition and plate) at 37 DPD. Each point represents the mean of one well across 5 minutes of recording. ** indicates $p<0.01$, ns indicates not significant. Paired t-test for comparison of each well to baseline measurement in TTX group, $t(17) = 2.9134$, $p = 0.009684$, and control group, $t(17) = -1.072$, $p = 0.2987$.

(H) Raster plots of firing rate at 37 DPD for one representative well, H9 line, at baseline and 5 minutes after addition of 1µM TTX. The raster plots show MFR in hertz (Hz, color bar) in 1-second bins for each electrode (rows) over 3 minutes scaled to the range of MFR in the entire dataset.

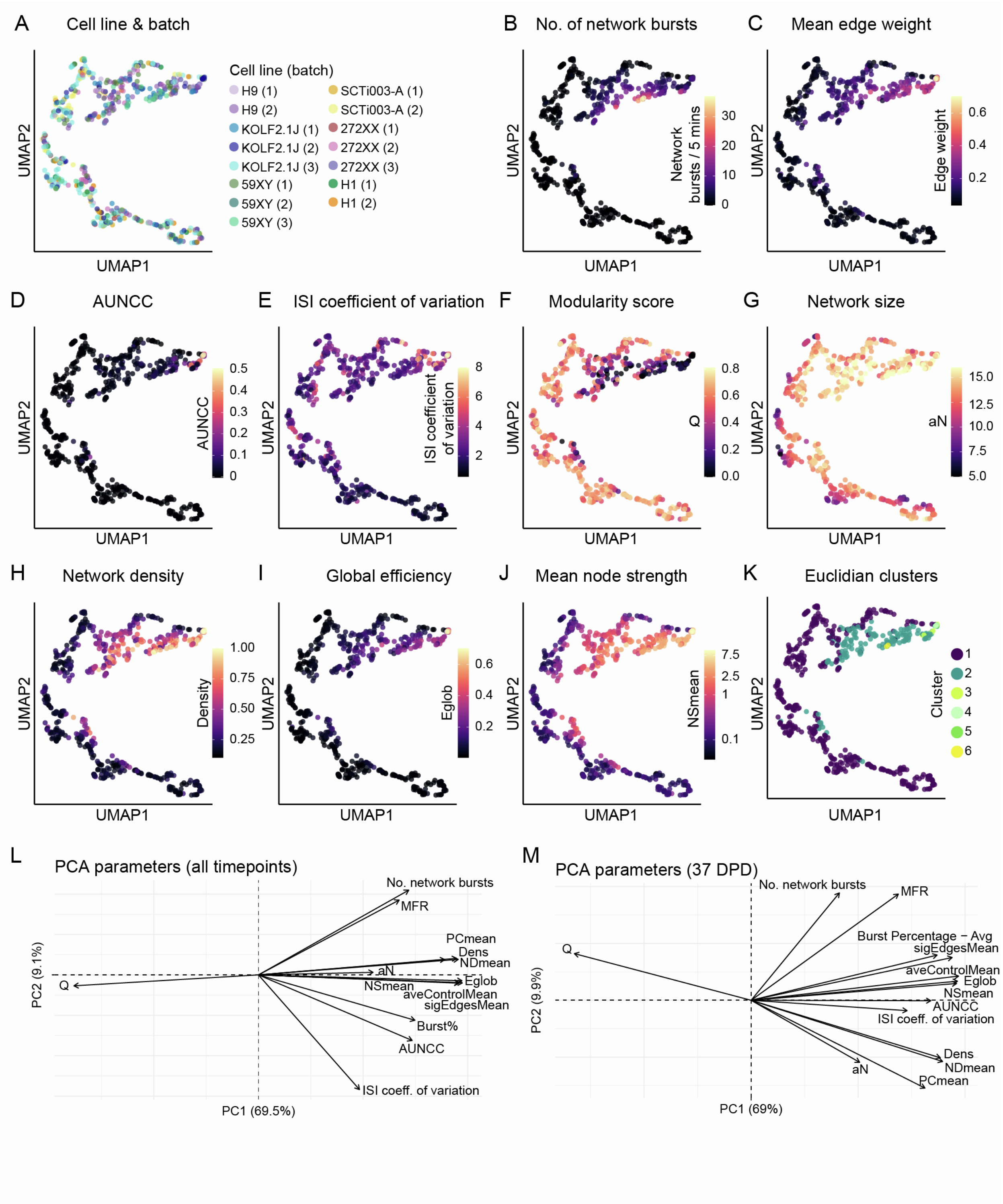

Figure S3. UMAP visualizations of additional parameters included in dimensionality reduction analysis, Related to Figure 3

(A-K) UMAP visualizations of MEA wells from 14 neuronal activity and network parameters showing all wells with active electrodes across five timepoints, 6-, 15-, 22-, 29-, and 37-DPD. Each point represents one well at one timepoint.

(L-M) Biplots showing the impact of each included MEA parameter on the top two principal components across five timepoints (L) and at 37 DPD (M).

Abbreviations: An, network size; AUNCC, area under normalized cross-correlation; Burst%, mean percentage of spikes in bursts; Dens, network density; Eglob, global efficiency; ISI coeff. of variation, inter-spike interval coefficient of variation; MFR, mean firing rate; NDmean, mean node degree; No. network bursts, number of network bursts; NSmean, mean node strength; Q, modularity score; SigEdgesMean, mean significant edge weight; PCmean, mean participation coefficient.

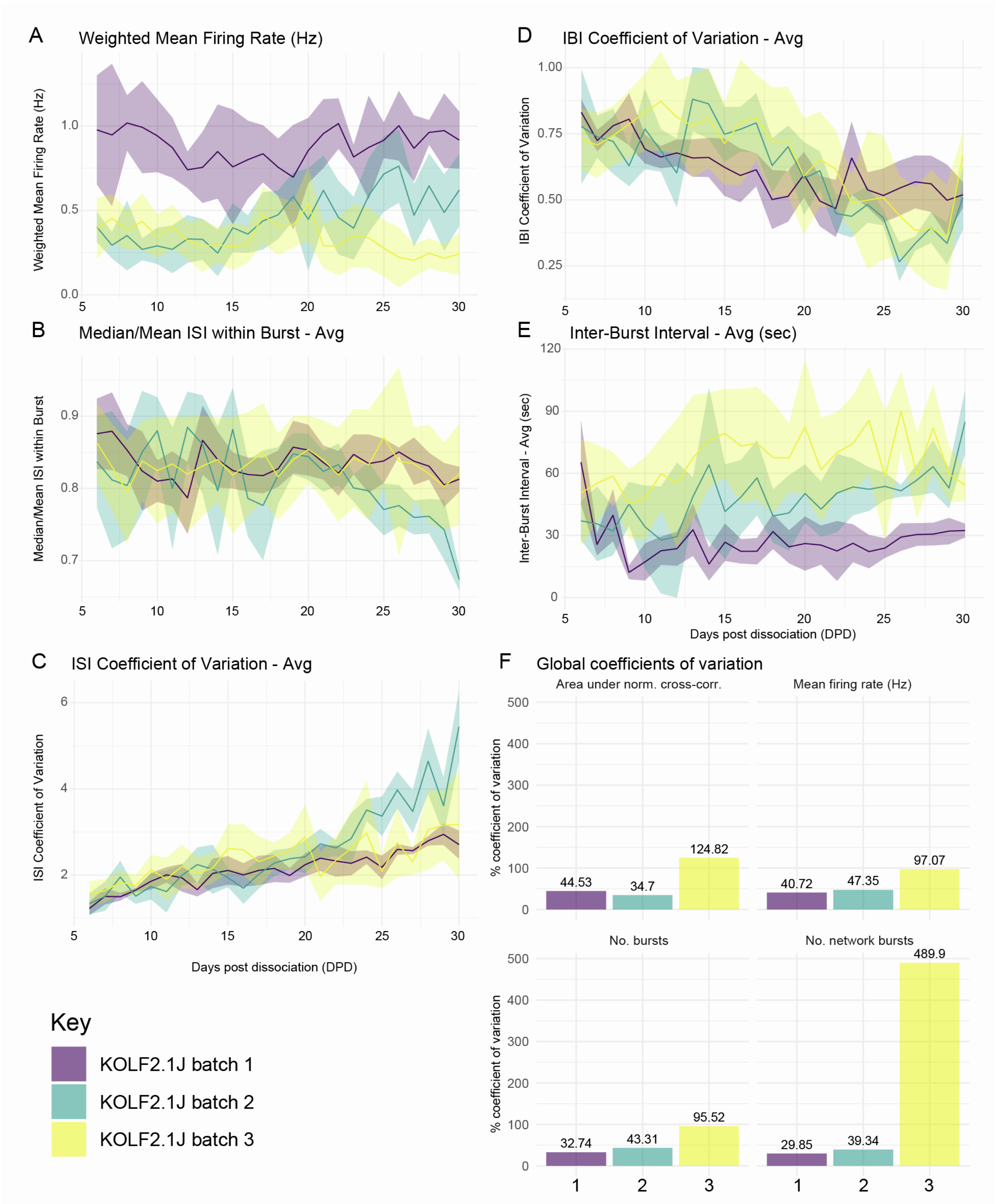

Figure S4. Development of spontaneous electrical activity and network properties vary between batches – additional parameters and coefficients of variation, related to Figure 4

(A-E) Line graphs of median across all wells for additional parameters over time showing the weighted mean firing rate (A), median/mean ISI within burst (B), ISI coefficient of variation (C), IBI coefficient of variation (D), IBI (E) across three independent batches of dissociated KOLF2.1J organoids, with one timepoint per day (5 mins recording). The ribbon represents the interquartile range.

(F) Bar graphs of “Global” coefficients of variation at 30 DPD for three batches of KOLF2.1J line dissociated organoids, for parameters shown in Figure 4. Coefficients of variation were calculated as the standard deviation of the well averages of each parameter divided by the mean of each parameter across all wells, multiplied by 100.

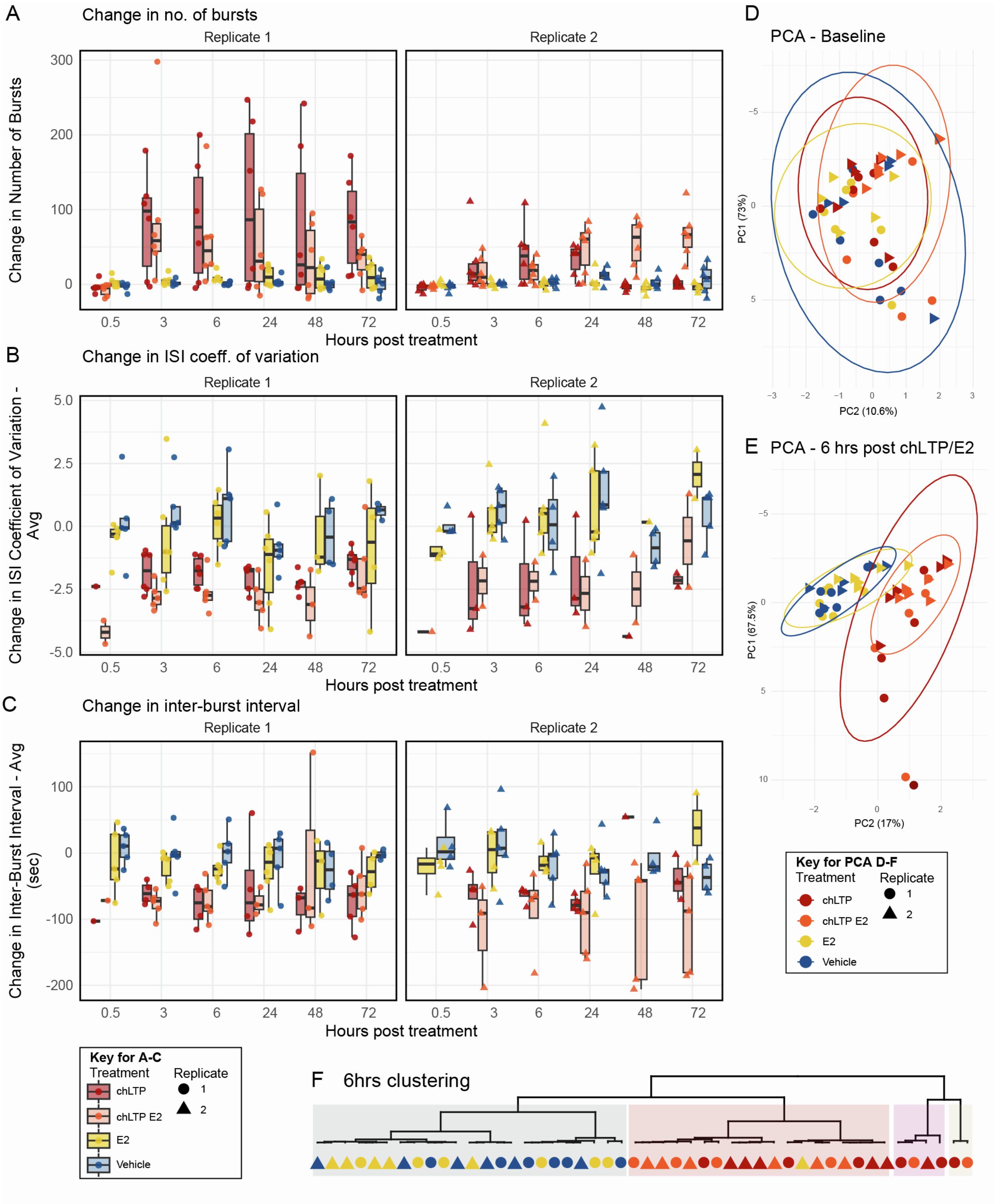

Figure S5. Acute 17β-estradiol does not affect electrical activity in dissociated organoids in combination with a chemical plasticity stimulus, related to Figure 6

(A-C) Boxplots of difference relative to baseline in MEA parameters after chLTP, chLTP+E2, E2, or vehicle treatment. Boxplots show the min, max, median, and interquartile range of the change in number of bursts / 5 minutes (A), ISI coefficient of variation (B), and inter-burst interval (C) per well, at six different

timepoints (0.5, 3, 6, 24, 48, 72 hours post treatment. Each point represents the mean of one well across 5 minutes of recording. The color of each point indicates the treatment (vehicle green, chLTP red, chLTP+E2 blue, E2 yellow). For each parameter, the left plot shows the first replicate and the plot on the right shows the second replicate. In B-C, only wells with bursts present are shown. Measured in the KOLF2.1J, with treatment starting at 34 DPD (79 DIV) for replicate one and 29 DPD (74 DIV) for replicate 2. The data for chLTP and Vehicle is also shown in Figure 6.

(D-E) Unsupervised clustering analysis of MEA wells shows a clear effect of chLTP but no effect of E2 exposure. PCA analysis of 10 MEA parameters measured at baseline (A) and measured 6 hours following chLTP and E2 pre-treatment (B). The PCA plots show the first and second principal components, representing the two main sources of variation in the data. Each point represents the one MEA well, colored by treatment. Point shape indicates the replicate. The data ellipses represent the 95% confidence interval, expected to enclose 95% of bivariate-normal t distributed data for each treatment condition.

(F) Dendrogram of hierarchical clustering on principal components analysis for each well at 6 hours following chLTP shows vehicle/E2 and chLTP/chLTP+E2 samples clustering together. Clustering done using Ward’s criterion for the first three principal components.

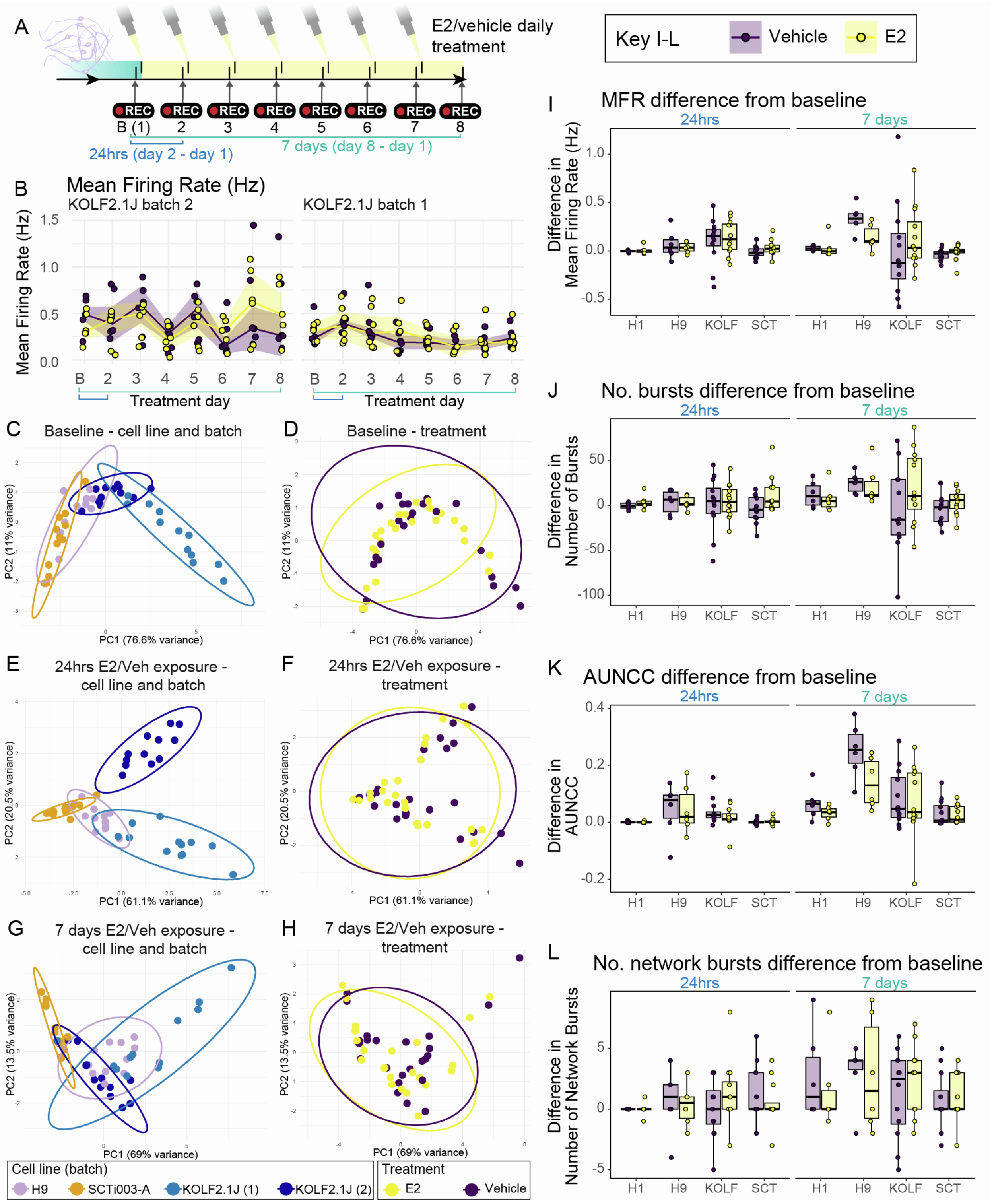

Figure S6. Long-term 17β-estradiol exposure does not affect electrical activity in dissociated organoids, related to Figure 6

(A) Schematic of treatment and recording timeline. The baseline recording was taken immediately prior to the first treatment on the first day. The next recording was taken 24 hours after the baseline, followed by

another treatment spike. Recordings and treatments in subsequent days were performed in the same manner.

(B) Line graph showing the median firing rate (B) across two independent batches of dissociated KOLF2.1J organoids, with one timepoint per day (5 mins recording). The ribbon represents the interquartile range. Each point represents one well. Wells without network bursting were excluded from the analysis. Point, line, and ribbon color indicates treatment (E2 yellow, vehicle purple). Treatment day 'B' is the baseline recording.

(C-H) Unsupervised clustering analysis of MEA wells shows batch and cell line differences but no effect of E2 exposure. Activity measured at baseline (C-D), 24hrs E2 exposure (E-F), and 7 days E2 exposure (G-H). PCA plots of the first and second principal components, representing the two main sources of variation in the data. Each point represents one MEA well, colored by cell line and batch (left, C, E , G) and treatment (right, D, F, H). The data ellipses represent the 95% confidence interval, expected to enclose 95% of bivariate-normal t distributed data for each cell line/batch (left) or treatment condition (right). Experiment performed in dissociated organoids across two independent batches from the KOLF21.J line and one batch for the H1 and STi003-A line starting at 29 DPD (baseline), dissociated at 45-47 DIV.

(I-L) Boxplots of the min, max, median, and interquartile range of the difference relative to baseline (day 1) in firing rate (I), number of bursts / 5 minutes (J), area under normalized cross- correlation (K), and number of network bursts / 5 minutes (L) per well after 24 hours or 7 days of daily treatment with E2/vehicle. Each point represents the difference in means of one well across 5 minutes of recording. The colors indicate the treatment. Experiment performed in dissociated organoids across two independent batches from the KOLF21.J and SCTi003-A line and one batch of the H1 and H9 line starting at 29 DPD (baseline). Statistical analysis in Table S3.

References for document S1

1. Axion BioSystems. (2021). Axis Navigator User Guide, 3.5 Edition (Axion BioSystems, Inc).
2. Sit, T.P.H., Feord, R.C., Dunn, A.W.E., Chabros, J., Oluigbo, D., Smith, H.H., Burn, L., Chang, E., Boschi, A., Yuan, Y., et al. (2024). MEA-NAP: A flexible network analysis pipeline for neuronal 2D and 3D organoid multielectrode recordings. Cell Reports Methods *4*, 100901. https://doi.org/10.1016/j.crmeth.2024.100901.
